# Robust Data-Reuse Regularized Recursive Least-Squares Algorithms for System Identification Applications †

**DOI:** 10.3390/s25165017

**Published:** 2025-08-13

**Authors:** Radu-Andrei Otopeleanu, Constantin Paleologu, Jacob Benesty, Laura-Maria Dogariu, Cristian-Lucian Stanciu, Silviu Ciochină

**Affiliations:** 1 Department of Telecommunications, National University of Science and Technology POLITEHNICA Bucharest, 060042 Bucharest, Romania; radu.otopeleanu@upb.ro (R.-A.O.); ldogariu@comm.pub.ro (L.-M.D.); cristian@comm.pub.ro (C.-L.S.); silviu@comm.pub.ro (S.C.); 2 Academy of Romanian Scientists, Ilfov 3, 050044 Bucharest, Romania; 3 INRS-EMT, University of Quebec, Montreal, QC H5A 1K6, Canada; jacob.benesty@inrs.ca

**Keywords:** adaptive filters, data-reuse, echo cancellation, recursive least-squares (RLS) algorithm, regularization, robustness, system identification

## Abstract

The recursive least-squares (RLS) algorithm stands out as an appealing choice in adaptive filtering applications related to system identification problems. This algorithm is able to provide a fast convergence rate for various types of input signals, which represents its main asset. In the current paper, we focus on the regularized version of the RLS algorithm, which also owns improved robustness in noisy conditions. Since convergence and robustness are usually conflicting criteria, the data-reuse technique is used to achieve a proper compromise between these performance features. In this context, we develop a computationally efficient approach for the data-reuse process in conjunction with the regularized RLS algorithm, using an equivalent single step instead of multiple iterations (for data-reuse). In addition, different regularization techniques are involved, which lead to variable-regularized algorithms, with time-dependent regularization parameters. This allows a better control in different challenging conditions, including noisy environments and other external disturbances. The resulting data-reuse regularized RLS algorithms are tested in the framework of echo cancellation, where the obtained results support the theoretical findings and indicate the reliable performance of these algorithms.

## 1. Introduction

There are many important real-world applications that rely on adaptive filters [1,2,3]. Such popular signal processing tools are frequently involved in system identification problems [4], interference cancellation schemes [5], channel equalization scenarios [6], sensor networks [7], and prediction configurations [8], among many others. The key block that controls the overall operation of this type of filter is the adaptive algorithm, which basically commands the coefficients’ update.

There are several families of adaptive filtering algorithms, among which two stand out as the most representative [1,2,3]. First, the least-mean-square (LMS) algorithms are popular due to their simplicity and practical features, especially in terms of low computational complexity. However, their performance is quite limited when operating with highly correlated input signals and/or long-length filters. The second important category of algorithms belong to the recursive least-squares (RLS) family, with improved convergence performance as compared to their LMS counterparts, even in the previously mentioned challenging scenarios. Nevertheless, the RLS algorithms are more computationally expensive and can also experience some stability problems in practical implementations. On the other hand, the performance of implementation platforms nowadays is exponentially improving, in terms of both processing speed and implementation facility. Consequently, the popularity of the RLS-type algorithms is also constantly growing, thus becoming the solution of choice in different frameworks [9,10,11].

Motivated by these aspects, the current paper targets further improvements on the global performance of the RLS algorithm, by aiming for a better control of its convergence parameters. The application framework focuses on the system identification problem, which represents one of the basic configurations in adaptive filtering, with a wide range of applications [2]. In this context, the forgetting factor represents one of the main parameters that tune the algorithm behavior [3]. This positive subunitary constant weights the square errors that contribute to the cost function, so that it mainly influences the memory of the algorithm. A larger value of this parameter (i.e., closer to one) leads to a better accuracy of the solution provided by the adaptive filter. However, in order to remain alert to any potential changes in the system to be identified, a lower value of the forgetting factor is desired, which would lead to a faster tracking reaction.

The RLS algorithm should also be robust to different external perturbations in the operating environment, which can frequently happen in system identification scenarios. For example, let us consider an echo cancellation context [5], where an acoustic sensor (i.e., microphone) captures the background noise from the surroundings, which can be significantly strong and highly nonstationary. In this case, the algorithm should be robust to such variations, a goal that cannot be achieved using only the forgetting factor as the control parameter. Toward this purpose, the cost function of the algorithm should include (besides the error-related term) an additional regularization component [12,13,14,15]. As a result, the robustness of the algorithm is controlled in terms of the resulting regularization parameter. Nevertheless, most of the regularized RLS algorithms require additional (a priori) information about the environment or need some extra parameters that are difficult to evaluate in practice. Moreover, a robust behavior to such external perturbations (related to the environment) is usually paid by a slower tracking reaction when dealing with time-varying systems.

These conflicting requirements, in terms of accuracy, tracking, and robustness, lead to a performance compromise between these main performance criteria. More recently, the data-reuse technique was also introduced and analyzed in the context of RLS algorithms [16,17,18]. The basic idea is to use the same set of data (i.e., the input and reference signals) several times within each main iteration of the algorithm, in order to improve the convergence rate and the tracking capability of the filter. Usually, mainly due to complexity reasons, the date-reuse method is extensively used in conjunction with LMS-type algorithms [19,20,21,22,23,24,25,26]. The solutions proposed in [16,18], in the framework of RLS-type algorithms, replace the multiple iterations of the data-reuse process with a single equivalent step, thus maintaining the computational complexity order of the original algorithm. Moreover, the data-reuse parameter (i.e., the number of equivalent data-reuse iterations) is used as an additional control factor, in order to improve the tracking capability of the RLS algorithm, even when operating with a large value of the forgetting factor.

The previous work [16] introduced the data-reuse principle in the context of the conventional RLS algorithm, which does not include any regularization component within its cost function, so that it is inherently limited in terms of robustness. Following [16], a convergence analysis of this algorithm was presented in [17]. More recently, we developed a data-reuse regularized RLS algorithm [18], with improved robustness features, where the regularization parameter is related to the signal-to-noise ratio (SNR). The current work represents an extension of the conference paper [18], with a twofold new contribution. First, it provides additional theoretical details and simulation results related to the algorithm developed in [18], also including a practical estimation of the SNR. Second, it presents a novel regularization technique recently proposed in [27], in conjunction with the data-reuse technique, thus resulting in a new RLS-type algorithm. Its regularization parameter considers both the influence of the external noise and a term related to the model’s uncertainties. This approach leads to improved performance as compared to the previously developed data-reuse regularized RLS algorithm.

Following this introduction, the rest of this paper is structured as follows. Section 2 contains the basics of the regularized RLS algorithms, including the recent method from [27]. Next, Section 3 develops the data-reuse method in conjunction with the regularized RLS algorithms. Simulation results are presented in Section 4, in the framework of echo cancellation. The paper is concluded in Section 5, which summarizes the main findings and outlines several perspectives for future research.

## 2. Regularized RLS Algorithms

Let us consider a system identification setup [3] having the reference (or desired) signal, obtained as

(1) $$\begin{array}{c} \begin{array}{cc} d(n) & =x^{T}\left(n\right)h\left(n\right)+v\left(n\right)\\ \; & =y(n)+v(n), \end{array} \end{array}$$

where *n* represents the discrete-time index,

$$\begin{array}{cc} x(n) & ={\left[\begin{array}{cccc} x(n) & x(n-1) & \cdots & x(n-L+1) \end{array}\right]}^{T} \end{array}$$

is a vector containing the most recent *L* samples of the zero-mean input signal x(n), with the superscript *^T^* denoting the transpose of a vector or a matrix,

$$\begin{array}{cc} h(n) & ={\left[\begin{array}{cccc} h_{0}\left(n\right) & h_{1}\left(n\right) & \cdots & h_{L-1}\left(n\right) \end{array}\right]}^{T} \end{array}$$

is the impulse response (of length *L*) of the system that we need to identify, and v(n) is a zero-mean additive noise signal, which is independent of x(n). In this context, the main objective is to identify h(n) with an adaptive filter, denoted by

$$\begin{array}{c} \hat{h}\left(n\right)={\left[\begin{array}{cccc} {\hat{h}}_{0}\left(n\right) & {\hat{h}}_{1}\left(n\right) & \cdots & {\hat{h}}_{L-1}\left(n\right) \end{array}\right]}^{T}. \end{array}$$

Therefore, the a priori error between the reference signal and its estimate results in

(2) $$\begin{array}{cc} e(n) & =d\left(n\right)-x^{T}\left(n\right)\hat{h}\left(n-1\right)\\ \; & =d\left(n\right)-\hat{y}\left(n\right), \end{array}$$

where $\hat{y}\left(n\right)$ represents the output of the adaptive filter. In the framework of echo cancellation [5], a replica of the echo signal is obtained at the output of the adaptive filter. In other words, the echo path impulse response is estimated/modeled by the adaptive filter, so that this unknown system (i.e., the echo path) is identified. Thus, the echo cancellation application can be formulated as a system identification problem.

This system identification problem can be solved following the least-squares (LS) optimization criterion [3], which is based on the minimization of the cost function:

(3) $$\begin{array}{cc} J(n) & =\sum_{i=1}^{n}\lambda^{n-i}{\left[d\left(i\right)-x^{T}\left(i\right)\hat{h}\left(n\right)\right]}^{2}, \end{array}$$

where λ is the forgetting factor, with 0<λ≤1. This parameter controls the memory of the algorithm, as explained in Section 1. The minimization of J(n) with respect to $\hat{h}\left(n\right)$ leads to the normal equations [3]:

(4) $$\begin{array}{cc} R\left(n\right)\hat{h}\left(n\right) & =c(n), \end{array}$$

where

(5) $$\begin{array}{cc} R(n) & =\lambda R\left(n-1\right)+x\left(n\right)x^{T}\left(n\right), \end{array}$$

(6) $$\begin{array}{cc} c(n) & =\lambda c(n-1)+x(n)d(n). \end{array}$$

The estimates from (5) and (6) are associated, respectively, to the covariance matrix of the input signal, and to the cross-correlation vector between the input and reference sequences. The system of equations from (4) can be recursively solved, thus leading to the conventional RLS algorithm [3], which is defined by the update

(7) $$\begin{array}{cc} \hat{h}\left(n\right) & =\hat{h}\left(n-1\right)+R^{-1}\left(n\right)x\left(n\right)e\left(n\right). \end{array}$$

The standard initialization of this algorithm is $\hat{h}\left(0\right)=0_{L}$ and $R\left(0\right)=\delta_{+}^{-1}I_{L}$, where $0_{L}$ and $I_{L}$ denote an all-zero vector of length *L* and the identity matrix of size L×L, respectively, while $\delta_{+}$ is a positive constant, also known as the regularization parameter. However, the influence of $\delta_{+}$ is only limited to the initial convergence of the algorithm, since its contribution is diminishing when *n* increases, due to the presence of the subunitary forgetting factor λ. As we can notice from (5), when using the previous initialization for R(0), the matrix R(n) contains the term $\lambda^{n}\delta_{+}I_{L}$, which is basically negligible for *n* large enough and λ<1. As a result, the overall performance of the conventional RLS algorithm, in terms of accuracy and tracking, is basically influenced by the forgetting factor, which represents the main control parameter. On the other hand, these performance criteria are conflicting, since a large value of λ (i.e., close to one) leads to a good accuracy of the filter estimate, but with a slow tracking reaction (when the system changes). In order to improve the tracking behavior, the forgetting factor should be reduced, while sacrificing the accuracy of the solution. In terms of robustness (against external perturbations), the higher the value of λ, the more robust the algorithm is. However, there is an inherent performance limitation even for λ=1, so that using the forgetting factor as the single control mechanism is not always a practical asset.

As outlined in Section 1, the robustness of RLS-type algorithms can be improved by incorporating a proper regularization component directly into the cost function. There are different approaches to this problem; however, the practical issues should also be taken into account. In other words, the resulting regularization term should be easy to control in practice, without requiring additional or a priori knowledge related to the system or the environment. Among the existing solutions, we present in the following two practical regularization techniques.

The first one involves the Euclidean norm (or the $l_{2}$ regularization), so that the cost function of the regularized RLS algorithm [1] results in

(8) $$\begin{array}{cc} J^{\prime }\left(n\right) & =\sum_{i=1}^{n}\lambda^{n-i}{\left[d\left(i\right)-x^{T}\left(i\right)\hat{h}\left(n\right)\right]}^{2}+\delta^{\prime }{∥\hat{h}\left(n\right)∥}^{2}, \end{array}$$

where $∥\cdot ∥$ denotes the Euclidean norm. In this case, the update of the regularized RLS algorithm becomes

(9) $$\begin{array}{cc} \hat{h}\left(n\right) & =\hat{h}\left(n-1\right)+{\left[R\left(n\right)+\delta^{\prime }I_{L}\right]}^{-1}x\left(n\right)e\left(n\right). \end{array}$$

In order to find a proper value of $\delta^{\prime }$, the solution proposed in [12] rewrites the update from (9) as

(10) $$\begin{array}{cc} \hat{h}\left(n\right) & =Q\left(n\right)\hat{h}\left(n-1\right)+\underline{\hat{h}}\left(n\right), \end{array}$$

where

(11) $$\begin{array}{cc} Q(n) & =I_{L}-{\left[R\left(n\right)+\delta^{\prime }I_{L}\right]}^{-1}x\left(n\right)x^{T}\left(n\right), \end{array}$$

(12) $$\begin{array}{cc} \underline{\hat{h}}\left(n\right) & ={\left[R\left(n\right)+\delta^{\prime }I_{L}\right]}^{-1}x\left(n\right)d\left(n\right). \end{array}$$

In this way, we can notice a “separation” in the right-hand side of (10), where Q(n) depends only on the input signal, while $\underline{\hat{h}}\left(n\right)$ represents the correctiveness component of the algorithm. In relation to this component, a new error signal can be defined as

(13) $$\begin{array}{cc} \underline{e}\left(n\right) & =d\left(n\right)-x^{T}\left(n\right)\underline{\hat{h}}\left(n\right). \end{array}$$

At this point, in order to attenuate the effects of the noise in the estimate from (12), the condition imposed in [12] is to find $\delta^{\prime }$ in such a way that

(14) $$\begin{array}{cc} E\left[{\underline{e}}^{2}\left(n\right)\right] & =\sigma_{v}^{2}, \end{array}$$

where $E\left[\cdot \right]$ stands for mathematical expectation and $\sigma_{v}^{2}=E\left[v^{2}\left(n\right)\right]$ is the variance of the noise signal from (1). Developing (14) based on (12), the regularization parameter results in [12]

(15) $$\begin{array}{cc} \delta^{\prime } & =L\frac{1+\sqrt{1+\mathrm{SNR}}}{\mathrm{SNR}}\sigma_{x}^{2}, \end{array}$$

where $\mathrm{SNR}=\sigma_{y}^{2}/\sigma_{v}^{2}$ represents the signal-to-noise ratio, while $\sigma_{y}^{2}=E\left[y^{2}\left(n\right)\right]$ and $\sigma_{x}^{2}=E\left[x^{2}\left(n\right)\right]$ are the variances of the output signal and the input sequence, respectively. It can be noticed from (15) that a low SNR leads to a high value of $\delta^{\prime }$ and, consequently, to a small update term in (9), which is the desired behavior in terms of robustness in noisy conditions. Nevertheless, in practice, the SNR is not available and should be estimated.

A simple yet efficient method to this purpose was proposed in [13]. It relies on the assumption that the adaptive filter has converged to a certain degree, i.e., $y\left(n\right)\approx \hat{y}\left(n\right)$, so that $\sigma_{y}^{2}\approx \sigma_{\hat{y}}^{2}$, where $\sigma_{\hat{y}}^{2}=E\left[{\hat{y}}^{2}\left(n\right)\right]$ denotes the variance of the estimated output from the right-hand side of (2). Also, since y(n) and v(n) are uncorrelated, taking the expectation in (1) results in

(16) $$\begin{array}{c} \begin{array}{cc} \sigma_{d}^{2} & =\sigma_{y}^{2}+\sigma_{v}^{2}\\ \; & \approx \sigma_{\hat{y}}^{2}+\sigma_{v}^{2}, \end{array} \end{array}$$

where $\sigma_{d}^{2}=E\left[d^{2}\left(n\right)\right]$ is the variance of the reference signal. Therefore, $\sigma_{v}^{2}\approx \sigma_{d}^{2}-\sigma_{\hat{y}}^{2}$, so that the SNR can be approximated as

(17) $$\begin{array}{cc} \mathrm{SNR} & \approx \frac{\sigma_{\hat{y}}^{2}}{\epsilon +|\sigma_{d}^{2}-\sigma_{\hat{y}}^{2}|}, \end{array}$$

where the absolute value at the denominator is used to prevent any minor deviations of the estimates (which could make the SNR negative) and ϵ is a very small positive constant that prevents a division by zero. Since the signals required in (17) are available, i.e., d(n) and $\hat{y}\left(n\right)$, their associated variances can be recursively estimated as

(18) $$\begin{array}{cc} \sigma_{d}^{2}\left(n\right) & =\lambda \sigma_{d}^{2}\left(n-1\right)+\left(1-\lambda \right)d^{2}\left(n\right), \end{array}$$

(19) $$\begin{array}{cc} \sigma_{\hat{y}}^{2}\left(n\right) & =\lambda \sigma_{\hat{y}}^{2}\left(n-1\right)+\left(1-\lambda \right){\hat{y}}^{2}\left(n\right), \end{array}$$

where the forgetting factor λ is now used as a weighting parameter. The initialization is $\sigma_{d}^{2}\left(0\right)=\sigma_{\hat{y}}^{2}\left(0\right)=0$. The resulting algorithm defined by the update (9), which uses $\delta^{\prime }$ computed based on (15) and using the estimated SNR from (17), is referred to as the variable-regularized RLS (VR-RLS) algorithm [13].

The second practical regularization technique analyzed in this work has been recently proposed in [27]. It considers a linear state model, where the observation equation is given in (1), while the state system follows a simplified first-order Markov model:

(20) $$\begin{array}{cc} h(n) & =h(n-1)+w(n), \end{array}$$

where w(n) is a zero-mean white Gaussian noise signal vector, which is uncorrelated to h(n−1) and v(n). Related to this model, we denote by $\sigma_{w}^{2}$ and $R_{w}\left(n\right)=\sigma_{w}^{2}I_{L}$ the variance and covariance matrix of w(n), respectively. The first-order Markov model is frequently used for modeling time-varying systems (or nonstationary environments), especially in the context of adaptive filters [1,2,3]. Moreover, this model fits very well for echo cancellation scenarios [5], where the impulse response of the echo path (to be modeled by the adaptive filter) is associated to a time-varying system, which can be influenced by several factors. For example, in acoustic echo cancellation, the room impulse response is influenced by temperature, pressure, humidity, and the movement of objects or bodies. Thus, the model in (20) represents a benchmark in this framework in order to model the unknown dynamics of the environment. In this context, it represents a particularly convenient stochastic model for such time-varying systems. This model represents systems that gradually change into an unpredictable direction, which is strongly in agreement with the nature of time-varying impulse responses of the echo paths.

Next, in order to find the estimate $\hat{h}\left(n\right)$, the weighted LS criterion is used, together with a regularization term that incorporates the model uncertainties, which are captured by $\sigma_{w}^{2}$. Consequently, the cost function is

(21) $$\begin{array}{c} \begin{array}{cc} J^{\prime \prime }\left(n\right) & =\sum_{i=1}^{n}\lambda^{n-i}\frac{{\left[d\left(n\right)-x^{T}\left(n\right)\hat{h}\left(n\right)\right]}^{2}}{\sigma_{v}^{2}}\\ \; & +\frac{1}{L}\sum_{i=1}^{n}\lambda^{n-i}{\left[\hat{h}\left(n\right)-h\left(i-1\right)\right]}^{T}R_{w}^{-1}\left(n\right)\left[\hat{h}\left(n\right)-h\left(i-1\right)\right]. \end{array} \end{array}$$

This cost function takes into consideration both types of noise, i.e., the external noise that corrupts the output of the system and the internal noise that models the system uncertainties. In (21), the first term consists of the standard RLS cost function from (3), which is weighted by the external noise power ($\sigma_{v}^{2}$), while the second term consists of a weighted sum (using the same forgetting factor λ) of the terms that contain the covariance matrix of w(n) in order to capture the model uncertainties ($\sigma_{w}^{2}$).

The minimization of $J^{\prime \prime }\left(n\right)$ with respect to $\hat{h}\left(n\right)$ leads to a set of normal equations that can be recursively solved using the update [27]:

(22) $$\begin{array}{cc} \hat{h}\left(n\right) & =\hat{h}\left(n-1\right)+{\left[R\left(n\right)+\delta^{\prime \prime }I_{L}\right]}^{-1}x\left(n\right)e\left(n\right), \end{array}$$

where

(23) $$\begin{array}{cc} \delta^{\prime \prime } & =\frac{1}{L(1-\lambda )}\cdot \frac{\sigma_{v}^{2}}{\sigma_{w}^{2}}. \end{array}$$

The second term from the right-hand side of (23) can be interpreted as the noise-to-uncertainty ratio (NUR) and captures the effects of both “noises,” i.e., the external perturbation and the model uncertainties, which are related to the environment conditions and system variability, respectively. Clearly, the NUR is unavailable in practice and should be estimated. The solution proposed in [27] uses the following recursive estimators for the main parameters required in (23):

(24) $$\begin{array}{cc} \sigma_{v}^{2}\left(n\right) & =\lambda \sigma_{v}^{2}\left(n-1\right)+\left(1-\lambda \right)e^{2}\left(n\right), \end{array}$$

(25) $$\begin{array}{cc} \sigma_{w}^{2}\left(n\right) & =\lambda \sigma_{w}^{2}\left(n-1\right)+\left(1-\lambda \right)\frac{{∥\hat{h}\left(n\right)-\hat{h}\left(n-1\right)∥}^{2}}{L}, \end{array}$$

using the same forgetting factor λ as a weighting parameter. The initialization is $\sigma_{v}^{2}\left(0\right)=0$ and $\sigma_{w}^{2}\left(0\right)=\xi$, where the small positive constant ξ is used since the estimate from (25) appears at the denominator of (23).

The estimator from (24) is based on the fact that in system identification scenarios, the goal of the adaptive algorithm is not to drive the error signal to zero, since this would introduce noise into the filter estimate. Instead, the noise signal should be recovered from the error of the adaptive filter after this one converges to its steady-state solution. In other words, some related information about v(n) can be extracted from the error signal, e(n). Second, the estimator from (25) is derived based on (20). Thus, using the adaptive filter estimates from time indices *n* and n−1, we can use the approximation $\hat{h}\left(n\right)-\hat{h}\left(n-1\right)\approx w\left(n\right)$, while ${∥w(n)∥}^{2}\approx L\sigma_{w}^{2}$, for L≫1. In this way, the term ${∥\hat{h}\left(n\right)-\hat{h}\left(n-1\right)∥}^{2}$ captures the uncertainties in the system. In summary, the resulting regularized RLS algorithm based on the weighted LS criterion, referred to as WR-RLS [27], is defined by the update (22), with the regularization parameter $\delta^{\prime \prime }$ evaluated as in (23), and using the estimated NUR based on (24) and (25).

## 3. Data-Reuse Regularized RLS Algorithms

The general update of the regularized RLS algorithms presented in the previous section can be summarized as follows:

(26) $$\begin{array}{cc} \hat{h}\left(n\right) & =\hat{h}\left(n-1\right)+P\left(n\right)x\left(n\right)e\left(n\right), \end{array}$$

where

(27) $$\begin{array}{c} P\left(n\right)={\left[R\left(n\right)+\delta I_{L}\right]}^{-1} \end{array}$$

and δ generally denotes the regularization parameter, which can be a positive constant or a variable term that can be evaluated, as in (15) or (23). The filter update from (26) is performed for each set of data, i.e., x(n) and d(n), and for each time index *n*. On the other hand, in the context of the data-reuse approach, this process is repeated *N* times for the same time index *n*, i.e., the same set of data is reused *N* times. As a result, for the regularized RLS algorithms, the relations that define the data-reuse are

$$\begin{array}{cc} \mathrm{Initialization}:\;\; & g_{0}\left(n\right)=\hat{h}\left(n-1\right)\\ \mathrm{Data}-\mathrm{reuse}:\;\; & \mathrm{for}\;j=1,2,\ldots ,N \end{array}$$

(28) $$\begin{array}{cc} \; & \varepsilon_{j}\left(n\right)=d\left(n\right)-x^{T}\left(n\right)g_{j-1}\left(n\right) \end{array}$$

(29) $$\begin{array}{cc} \; & g_{j}\left(n\right)=g_{j-1}\left(n\right)+P\left(n\right)x\left(n\right)\varepsilon_{j}\left(n\right)\\ \mathrm{Update}:\;\; & \hat{h}\left(n\right)=g_{N}\left(n\right). \end{array}$$

Since P(n) [or R(n)] depends only on x(n), it remains the same within the cycle associated with the data-reuse process. It can be noticed that the conventional regularized RLS algorithm from (26) is obtained when N=1.

Nevertheless, it is not efficient (especially in terms of computational complexity) to implement the data-reuse process in the conventional way, as presented before. As an equivalent alternative, we show in the following how the entire data-reuse cycle can be efficiently grouped into a single update of the filter. Let us begin with the first step, which can be written as

(30) $$\begin{array}{cc} \varepsilon_{1}\left(n\right) & =d\left(n\right)-x^{T}\left(n\right)g_{0}\left(n\right), \end{array}$$

(31) $$\begin{array}{cc} g_{1}\left(n\right) & =g_{0}\left(n\right)+P\left(n\right)x\left(n\right)\varepsilon_{1}\left(n\right). \end{array}$$

The previous relations are then involved within the second step, which can be developed as

(32) $$\begin{array}{c} \begin{array}{cc} \varepsilon_{2}\left(n\right) & =d\left(n\right)-x^{T}\left(n\right)g_{1}\left(n\right)\\ \; & =d\left(n\right)-x^{T}\left(n\right)\left[g_{0}\left(n\right)+P\left(n\right)x\left(n\right)\varepsilon_{1}\left(n\right)\right]\\ \; & =d\left(n\right)-x^{T}\left(n\right)g_{0}\left(n\right)-x^{T}\left(n\right)P\left(n\right)x\left(n\right)\varepsilon_{1}\left(n\right)\\ \; & =\varepsilon_{1}\left(n\right)-q\left(n\right)\varepsilon_{1}\left(n\right)\\ \; & =r\left(n\right)\varepsilon_{1}\left(n\right), \end{array} \end{array}$$

using the notation:

(33) $$\begin{array}{cc} q(n) & =x^{T}\left(n\right)P\left(n\right)x\left(n\right), \end{array}$$

(34) $$\begin{array}{cc} r(n) & =1-q(n). \end{array}$$

It was also taken into account that $P\left(n\right)=P^{T}\left(n\right)$, due to the specific symmetry of the matrix R(n). Therefore, in this second step, we can evaluate the update of the filter as

(35) $$\begin{array}{c} \begin{array}{cc} g_{2}\left(n\right) & =g_{1}\left(n\right)+P\left(n\right)x\left(n\right)\varepsilon_{2}\left(n\right)\\ \; & =g_{0}\left(n\right)+P\left(n\right)x\left(n\right)\varepsilon_{1}\left(n\right)+P\left(n\right)x\left(n\right)r\left(n\right)\varepsilon_{1}\left(n\right)\\ \; & =g_{0}\left(n\right)+\left[1+r(n)\right]P\left(n\right)x\left(n\right)\varepsilon_{1}\left(n\right). \end{array} \end{array}$$

Similarly, the third step of the data-reuse process becomes equivalent to

(36) $$\begin{array}{cc} \varepsilon_{3}\left(n\right) & =r^{2}\left(n\right)\varepsilon_{1}\left(n\right), \end{array}$$

(37) $$\begin{array}{cc} g_{3}\left(n\right) & =g_{0}\left(n\right)+\left[1+r\left(n\right)+r^{2}\left(n\right)\right]P\left(n\right)x\left(n\right)\varepsilon_{1}\left(n\right). \end{array}$$

Following the same approach and using mathematical induction, we obtain the relations associated with the final *N*th step of the cycle, i.e.,

(38) $$\begin{array}{cc} \varepsilon_{N}\left(n\right) & =r^{N-1}\left(n\right)\varepsilon_{1}\left(n\right), \end{array}$$

(39) $$\begin{array}{cc} g_{N}\left(n\right) & =g_{0}\left(n\right)+\sum_{l=0}^{N-1}r^{l}\left(n\right)P\left(n\right)x\left(n\right)\varepsilon_{1}\left(n\right). \end{array}$$

It is known that $\varepsilon_{1}\left(n\right)=e\left(n\right)$, $g_{0}\left(n\right)=\hat{h}\left(n-1\right)$, $g_{N}\left(n\right)=\hat{h}\left(n\right)$, and $\sum_{l=0}^{N-1}r^{l}\left(n\right)$ sums the *N* terms of a geometric progression with the common ratio r(n). The later term can be computed as

(40) $$\begin{array}{cc} s(n) & =\frac{1-r^{N}\left(n\right)}{1-r(n)}, \end{array}$$

so that the final update becomes

(41) $$\begin{array}{cc} \hat{h}\left(n\right) & =\hat{h}\left(n-1\right)+s\left(n\right)P\left(n\right)x\left(n\right)e\left(n\right). \end{array}$$

**Figure 1:**
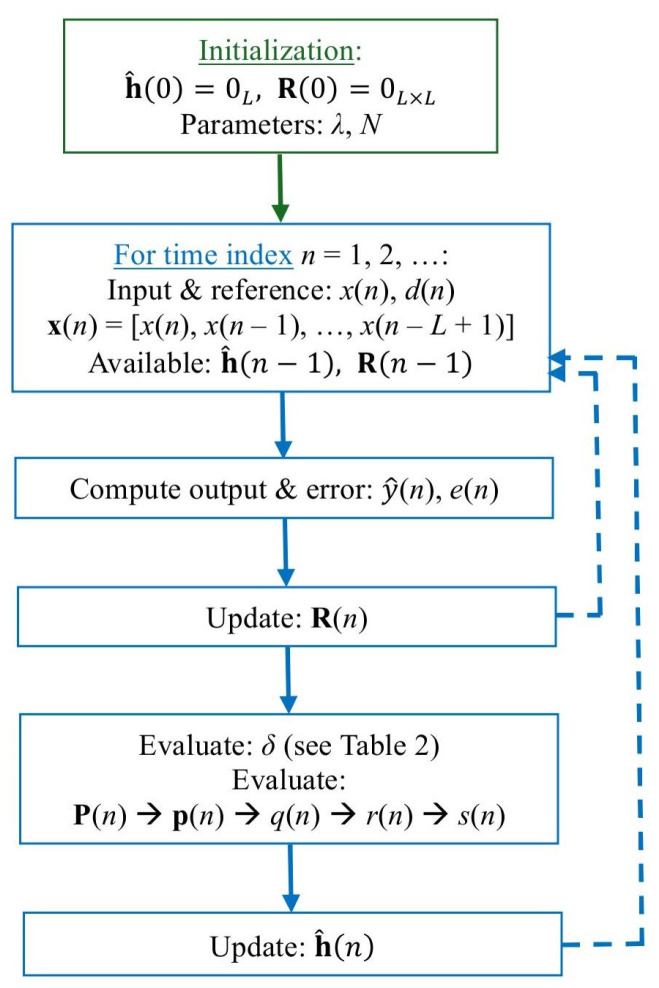
Block diagram of the data-reuse regularized RLS algorithm from Table 1.

The resulting data-reuse regularized RLS algorithm is summarized in Table 1 in a slightly modified form, which targets a more efficient implementation. Also, a block diagram of this type of algorithm is presented in Figure 1. The most challenging operations (in terms of complexity) are the matrix inversion and the computation of p(n). However, these steps could be efficiently solved using line search methods, like the conjugate gradient (CG) or coordinate descent (CD) algorithms [28,29,30]. (These methods have not been considered here for the implementation of the data-reuse regularized RLS-type algorithms, since they are beyond the scope of this paper. However, they represent a subject for future work, as will be outlined in Section 5). Also, the update of R(n) can be computed by taking into account the symmetry of this matrix and the time-shift property of the input vector, x(n). Thus, only the first row and column should be computed, while the rest of the elements are available from the previous iteration. As compared to the conventional regularized RLS algorithm, there is only a moderate increase in terms of computational complexity, mainly due to the evaluation of $q\left(n\right)=x^{T}\left(n\right)p\left(n\right)$. Nevertheless, this extra computational amount is reasonable, i.e., *L* multiplications and L−1 additions.

The data-reuse parameter *N* can play the role of an additional control factor, besides the forgetting factor λ. In this context, we aim to improve the overall performance of the algorithm, even when using a very large value of λ (i.e., very close or equal to 1), which leads to a good accuracy, but significantly affects the tracking. As a consequence, the data-reuse regularized RLS algorithm can attain a better compromise between the main performance criteria, i.e., accuracy versus tracking. Moreover, using a proper regularization parameter for this type of algorithm, like in (15) or (23), can improve its behavior in noisy environments.

The regularization parameter of the data-reuse regularized RLS algorithm can be set or evaluated in different ways, as indicated in Table 2. In the simplest approach, δ is selected as a positive constant, thus resulting in the data-reuse conventionally regularized RLS (DR-CR-RLS) algorithm. A more rigorous method for setting the constant regularization parameter relies on its connection to the SNR [12]. In conjunction with the data-reuse technique, this led to the data-reuse optimally regularized RLS (DR-OR-RLS) presented in [18].

Nevertheless, the estimated SNR from (17) was not considered in [18], where the true value of the SNR was assumed to be available in the evaluation of the “optimal” regularization constant, $\delta_{o}$ (see Table 2). As an extension to this previous work, the estimated SNR from (17) is considered in the current paper. Here, the parameter $\delta^{\prime }$ from (15) is used within the matrix P(n) from (41), but it is evaluated based on (17), in conjunction with (18) and (19). The resulting algorithm is referred to as the data-reuse variable-regularized RLS (DR-VR-RLS) algorithm. For N=1, it is equivalent to the VR-RLS algorithm from [13].

The regularization parameter specific to the WR-RLS algorithm [27], i.e., $\delta^{\prime \prime }$ from (23), can also be involved within the matrix P(n). Thus, in conjunction with the previously developed data-reuse process, which led to the update (41), a data-reuse WR-RLS (DR-WR-RLS) algorithm is obtained. Its regularization relies on (23), while using the estimated NUR based on (24) and (25). Also, the WR-RLS algorithm from [27] is a special case obtained when N=1.

As compared to the DR-VR-RLS algorithm that relies only on the estimated SNR, the regularization approach behind the DR-WR-RLS algorithm is potentially better, since it also includes the contribution of the model uncertainties within the NUR. In other words, the NUR can represent a better measure (for robustness control) instead of the SNR. For both DR-VR-RLS and DR-WR-RLS algorithms, their regularization parameters are time-dependent, so that the time index is indicated in Table 2, for $\delta^{\prime }\left(n\right)$ and $\delta^{\prime \prime }\left(n\right)$, respectively.

The computational complexity of these data-reuse regularized RLS algorithms is provided in Table 3 (in terms of the number of multiplications per iteration), as compared to the standard RLS and LMS algorithms [1,2,3]. Clearly, the LMS algorithm is the least complex, since its update is similar to (7), but using a positive constant μ (known as the step-size parameter) instead of $R^{-1}\left(n\right)$. However, it is known that the overall performance of the LMS algorithms (in terms of both the convergence rate and accuracy of the estimate) is inferior to the RLS-based algorithms, especially when operating with long-length filters and correlated input signals [1,2,3]. The complexity order of the RLS-based algorithms is proportional to $O(L^{2})$, but it also depends on the computational amount required by the matrix inversion, which is denoted by $O_{{•}^{-1}}$ in Table 3. The conventional RLS algorithm avoids this direct operation by using the matrix inversion lemma [3], so that its complexity order remains proportional to $O(L^{2})$. There are several alternative (iterative) techniques that can be used in this context, for solving the normal equations related to the RLS-based algorithms. Among the existing solutions, the dichotomous coordinate descent (DCD) method [28] represents one of the most popular choices, since it reduces the computational amount up to O(L), using a proper selection of its parameters. Nevertheless, the influence of these methods on the overall performance of the algorithms is beyond the scope of this paper and it will be investigated in future works (as will be outlined later in Section 4.6). The evaluation of the regularization parameter of the DR-⋆R-RLS algorithms from Table 2 (where ⋆ generally denotes the corresponding version, i.e., C/O/V/W), denoted by $O_{\delta }$, requires only a few operations, as compared to the overall amount. For example, in case of the DR-CR-RLS and DR-OR-RLS algorithms, the regularization parameters can be set a priori. The DR-VR-RLS requires only 6 multiplications per iteration for evaluating $\delta^{\prime }\left(n\right)$, while the computational amount related to $\delta^{\prime \prime \prime }\left(n\right)$ of the DR-WR-RLS algorithm is 6+L multiplications per iteration. Even if the DR-WR-RLS algorithm is the most complex among its counterparts from Table 2, its improved performance compensates for this extra computational amount, as will be supported in Section 4. Also, since N≪L, the computational amount required by the data-reuse process is negligible in the context of the overall complexity of the data-reuse regularized RLS algorithms. In addition, their robustness features justify the moderate extra computational amount as compared to the standard RLS algorithm.

Finally, we should outline that a detailed theoretical convergence analysis of the proposed algorithms is a self-containing issue that is beyond the scope of the paper and it will be explored in future works. Nevertheless, at the end of this section, we provide a brief convergence analysis in the mean value, under some simplified assumptions. First, let us consider that the covariance matrix of the input signal is close to a diagonal one, i.e., $E\left[x\left(n\right)x^{T}\left(n\right)\right]\approx \sigma_{x}^{2}I_{L}$. Consequently, for large enough *n*, its estimate from (5) results in $R\left(n\right)\approx \sigma_{x}^{2}/\left(1-\lambda \right)I_{L}$. Also, for L≫1 (like in echo cancellation scenarios), the approximation $x^{T}\left(n\right)x\left(n\right)\approx L\sigma_{x}^{2}$ is valid. At this point, let us note that a general rule for setting the forgetting factor is [18]

(42) $$\begin{array}{c} \lambda =1-\frac{1}{KL}, \end{array}$$

with K≥1. Under these circumstances, based on (27) and (34), we obtain

(43) $$\begin{array}{cc} P(n) & \approx \frac{1-\lambda }{\delta \left(1-\lambda \right)+\sigma_{x}^{2}}I_{L}=\frac{1}{\delta +KL\sigma_{x}^{2}}I_{L}, \end{array}$$

(44) $$\begin{array}{cc} r(n) & \approx 1-\frac{\left(1-\lambda \right)L\sigma_{x}^{2}}{\delta \left(1-\lambda \right)+\sigma_{x}^{2}}=1-\frac{L\sigma_{x}^{2}}{\delta +KL\sigma_{x}^{2}}. \end{array}$$

Since δ>0, it can be noticed that 0<r(n)<1 and s(n) can be considered as deterministic, being obtained as the sum of a geometric progression with *N* terms and the common ratio r(n). At the limit, when λ=1 (i.e., K→∞), the common ratio becomes r(n)=1, which results in s(n)=N.

Next, we assume that the system to be identified is time-invariant, so that its impulse response is fixed (for the purpose of this simplified analysis), i.e., h(n)≈h. In this context, the system mismatch (or the coefficients’ error) can be defined as $m\left(n\right)=h-\hat{h}\left(n\right)$, so that the condition for the convergence in the mean value results in $E\left[m(n)\right]=0_{L}$, for n→∞. This is equivalent to $E\left[\hat{h}\left(n\right)\right]=h$, for n→∞, which implies that the coefficients of the adaptive filter converge to those of the system impulse response. Based on (1) and (2), the update from (41) can be developed as

(45) $$\begin{array}{cc} \hat{h}\left(n\right) & =\hat{h}\left(n-1\right)+s\left(n\right)P\left(n\right)x\left(n\right)\left[x^{T}\left(n\right)m\left(n-1\right)+v\left(n\right)\right], \end{array}$$

so that subtracting h from both sides (and changing the sign), an update for the system mismatch is obtained as

(46) $$\begin{array}{cc} m(n) & =\left[I_{L}-s\left(n\right)P\left(n\right)x\left(n\right)x^{T}\left(n\right)\right]m\left(n-1\right)-s\left(n\right)P\left(n\right)x\left(n\right)v\left(n\right). \end{array}$$

Then, taking the expectation on both sides of (46), using (43), and considering that $E\left[x(n)v(n)\right]=0_{L}$ (since the input signal and the additive noise are uncorrelated), we obtain

(47) $$\begin{array}{cc} E\left[m(n)\right] & =\left[1-s\left(n\right)\frac{\sigma_{x}^{2}}{\delta +KL\sigma_{x}^{2}}\right]E\left[m(n-1)\right]. \end{array}$$

As indicated in Table 1, the initialization for the adaptive filter is $\hat{h}\left(n\right)=0_{L}$, so that m(0)=h. Hence, processing (47)—starting with this initialization—results in

(48) $$\begin{array}{cc} E\left[m(n)\right] & ={\left[1-s\left(n\right)\frac{\sigma_{x}^{2}}{\delta +KL\sigma_{x}^{2}}\right]}^{n}E\left[m(0)\right]\\ \; & ={\left[1-s\left(n\right)\frac{\sigma_{x}^{2}}{\delta +KL\sigma_{x}^{2}}\right]}^{n}h. \end{array}$$

Thus, to obtain the exponential decay toward zero, the convergence condition translates into $s\left(n\right)\sigma_{x}^{2}/\left(\delta +KL\sigma_{x}^{2}\right)<1$. Using the upper limit s(n)=N, which, as explained before, is related to (44), we need to verify that $N\sigma_{x}^{2}/\left(\delta +KL\sigma_{x}^{2}\right)<1$. Since δ>0, we have $N\sigma_{x}^{2}/\left(\delta +KL\sigma_{x}^{2}\right)<N\sigma_{x}^{2}/\left(KL\sigma_{x}^{2}\right)$, so that we basically need to verify that N/(KL)<1, i.e., N<KL. This condition is always true in practice, since the common setting is N≪L. Consequently, the data-reuse regularized RLS algorithm is convergent in the mean value.

In addition, a simple and reasonable mechanism to evaluate the stability of the algorithm is related to the conversion factor [1,3]. First, similarly to (2) but using the coefficients from the time index *n*, we can define the a posteriori error of the adaptive filter as

(49) $$\begin{array}{cc} \tilde{e}\left(n\right) & =d\left(n\right)-x^{T}\left(n\right)\hat{h}\left(n\right). \end{array}$$

Next, using the updated (41) in (49) and taking (2) into account, we obtain

(50) $$\begin{array}{cc} \tilde{e}\left(n\right) & =\left[1-s\left(n\right)x^{T}\left(n\right)P\left(n\right)x\left(n\right)\right]e\left(n\right)\\ \; & =\gamma (n)e(n), \end{array}$$

where

(51) $$\begin{array}{cc} \gamma (n) & =1-s\left(n\right)x^{T}\left(n\right)P\left(n\right)x\left(n\right) \end{array}$$

represents the so-called conversion factor. Under the same simplified assumptions used before (related to the convergence in the mean), this conversion factor can be approximated as

(52) $$\begin{array}{cc} \gamma (n) & \approx 1-\frac{NL\sigma_{x}^{2}}{\delta +KL\sigma_{x}^{2}}. \end{array}$$

For stability, we need to verify that 0<γ≤1, which further leads to $|\tilde{e}\left(n\right)|\leq |e\left(n\right)|$. Since the second term from the right-hand side of (52) is positive, the condition γ≤1 is always true. In order to also have γ>0, the ratio from (52) should be subunitary, i.e., $NL\sigma_{x}^{2}<\delta +KL\sigma_{x}^{2}$. Thus, using K≥N is sufficient to fulfill this condition and to guarantee the stability of the algorithm. This represents a common practical setting in most of the scenarios, as shown in the next section.

## 4. Simulation Results

The performances of the data-reuse regularized RLS algorithms presented in the previous section are analyzed in the following, based on the experiments performed in the framework of echo cancellation [5]. This type of application represents a very challenging system identification scenario, due to several main reasons. First, the input signal coming from the far-end (e.g., speech/audio or different type of noises) is usually nonstationary and also highly correlated. Second, the length of the system to be identified (i.e., the echo path) can be on the order of hundreds of coefficients. Third, the acoustic sensor (i.e., the microphone) that contains the echo signal also captures the background noise, together with the near-end voice and/or other external signals.

The previous factors raise significant challenges for the adaptive filtering algorithm, especially in terms of its convergence rate, tracking ability, accuracy of the estimate, and robustness to external (noisy) conditions. These represent the main performance criteria for assessing the overall behavior of the algorithms. As mentioned in Section 1, several robust RLS-based algorithms can be found in the literature. However, most of them are difficult to control in practice, since they usually require a priori information related to the operating environment and/or need the tuning of some additional parameters. Due to these reasons, the VR-RLS algorithm from [13] is considered a practical benchmark. The motivation behind this choice is twofold. First, it outperforms other robust RLS-based versions; second, it is considered a “practical” algorithm in terms of estimating/tuning its parameters in a facile manner. Clearly, the data-reuse technique presented in Section 3 can be applied to other robust RLS algorithms, but their previously mentioned limitations (related to the evaluation of the regularization term) still remain. For N=1, the DR-VR-RLS version is equivalent to the VR-RLS algorithm from [13]. In terms of the computational cost, most of the robust/regularized RLS-based algorithms are comparable, since the main computational amount is related to the relations that define the RLS part of the algorithm, while the evaluation of their regularization parameter contributes with only a few operations, as outlined in Table 3 in Section 3. Here, the proposed data-reuse regularized RLS algorithms are gradually discussed and compared in order to outline their main performance features and the practical aspects related to the regularization terms.

In the following, the experimental conditions are presented in Section 4.1. Then, in Section 4.2, Section 4.3, Section 4.4 and Section 4.5, the data-reuse regularized algorithms from Section 3 are gradually introduced and analyzed. Finally, a brief discussion is provided in Section 4.6.

### 4.1. Simulation Setup

The experiments involve two types of input signals, x(n), considering both stationary and nonstationary sequences. First, an autoregressive (AR) process is involved in simulations, which is obtained by filtering a white Gaussian noise through the transfer function $1/(1-0.9z^{-1})$. This first-order AR process, referred as AR(1), represents a stationary signal, but a highly correlated one, due to the pole (of the transfer function) close to unity. Second, a female voice is used as input, which is a more challenging signal due to its nonstationarity. The sampling frequency is 8 kHz.

The system to be identified is chosen according to the ITU-T G168 Recommendation [31]. It is based on the fourth cluster of coefficients ($b_{4}$), which has the length L=128. The impulse response of the echo path is obtained as $h\left(n\right)=b_{4}+u\left(n\right)$, where u(n) is a white Gaussian noise with the variance $\sigma_{u}^{2}$. This parameter is mainly set to $\sigma_{u}^{2}=10^{-4}{∥b_{4}∥}^{2}$, but it changes to $\sigma_{u}^{2}=10^{-2}{∥b_{4}∥}^{2}$ in several experiments in which an echo path change scenario is simulated at some point.

**Figure 2:**
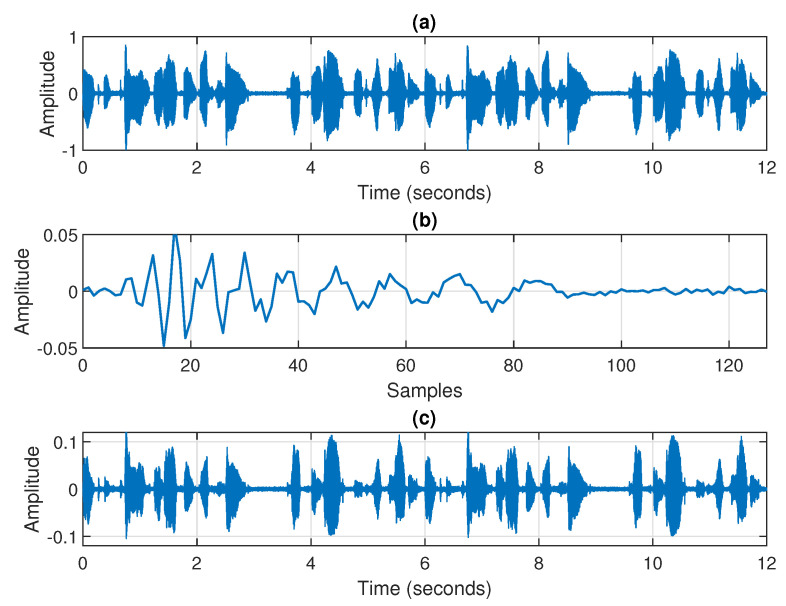
A set of input/output signal waveforms and the system impulse response used in simulations: (**a**) the input signal (the far-end speech), (**b**) the impulse response of the echo path, and (**c**) the output signal (the echo).

The output of the echo path, i.e., the echo signal, y(n), is corrupted by a white Gaussian noise, v(n), such that SNR=20 dB in most of the scenarios. Nevertheless, other perturbations are also considered in several experiments, like lower SNRs, different types of noise, and double-talk periods. These particular scenarios will be detailed in relation to each specific experiment from the following subsections. A set of input/output signal waveforms and the system impulse response used in simulations are depicted in Figure 2. This plot shows the far-end speech (i.e., the input signal), the system impulse response (i.e., the echo path), and the resulting echo (i.e., the output signal).

All the RLS-based algorithms are using the forgetting factor λ into their cost functions. This positive subunitary parameter is usually associated to the filter length, i.e., the longer the filter, the larger the λ value should be, as indicated in (42). In other words, the value of λ can be controlled in terms of the value of *K*, so that increasing this parameter results in increasing the forgetting factor. Also, as we can notice in (42), a larger value of *L* involves a larger value of λ, i.e., closer to one. The specific selection of λ (or *K*) will be indicated in each of the following experiments. The positive constant that appears within the denominator of several relations in order to prevent a division by zero (see Table 2) is set to $\epsilon =10^{-5}$. As a performance measure used to assess the behavior of all the analyzed algorithms, the normalized misalignment (in dB) is defined as

(53) $$\begin{array}{c} \mathrm{NM}\left[h\left(n\right),\hat{h}\left(n\right)\right]=20\log_{10}\frac{∥h\left(n\right)-\hat{h}\left(n\right)∥}{∥h(n)∥}. \end{array}$$

This represents one of the most popular indicators to evaluate the system identification scenarios, since it basically focuses on the “difference” (computed based on the Euclidean norm) between the true impulse response and its estimate. A lower value of $\mathrm{NM}\left[h\left(n\right),\hat{h}\left(n\right)\right]$ indicates a more accurate solution. Also, a steeper misalignment curve is associated to a faster convergence rate and/or a better tracking capability.

### 4.2. The DR-CR-RLS Algorithm

In the first set of experiments, we assess the performance of the DR-CR-RLS algorithm, which represents the conventional benchmark that uses a constant value for its regularization parameter, as indicated in the beginning of Table 2. The input signal is an AR(1) process and SNR=20 dB. The constant regularization term is set to $\delta =20\sigma_{x}^{2}$, which represents a general rule of thumb, as outlined in [12] and also explained later at the end of Section 4.3.

**Figure 3:**
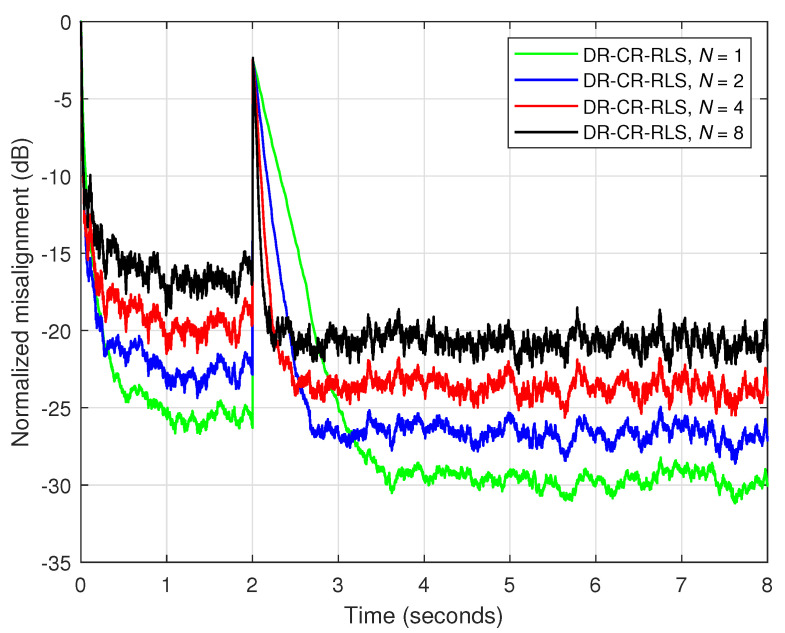
Normalized misalignment of the DR-CR-RLS algorithm using different values of the data-reuse parameter *N*. The other settings are λ=1−1/(20L) and $\delta =20\sigma_{x}^{2}$. The input signal is an AR(1) process, SNR=20 dB, L=128, and the echo path changes after 2 s.

**Figure 4:**
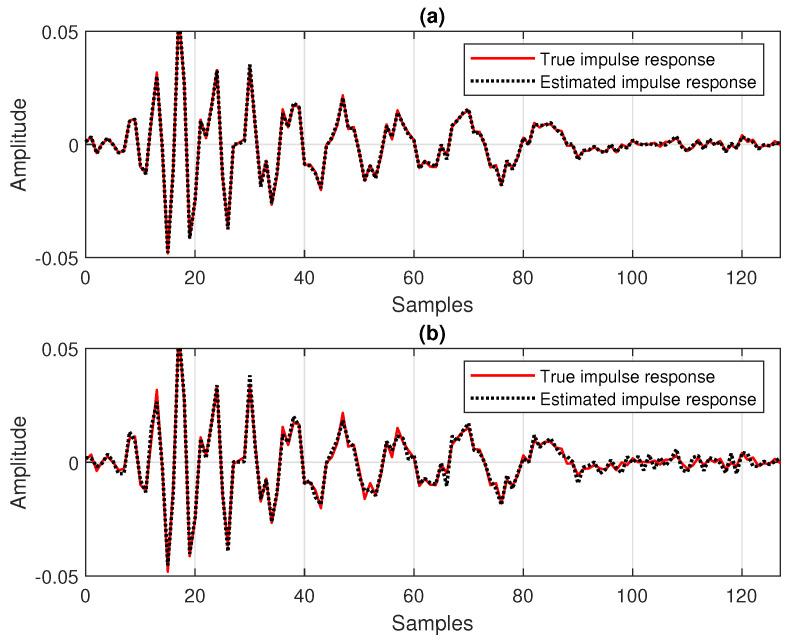
Estimated impulse response of the echo path (before the change), as compared to the true impulse response, for different values of the data-reuse parameter *N*, in relation to the experiment from Figure 3: (**a**) N=1 and (**b**) N=8.

Under these circumstances, in Figure 3, an echo-path-change scenario is simulated after 2 s. The results from Figure 3 illustrate the influence of the data-reuse parameter (*N*) on the overall performance of the DR-CR-RLS algorithm for a fixed forgetting factor, which is set to λ=1−1/(20L). Also, the estimated echo path (before the change), as compared to its true impulse response, is shown in Figure 4 for two values of *N*. It can be noticed from Figure 3 that a larger value of *N* leads to a faster tracking reaction when the echo path changes. On the other hand, this gain is paid in terms of accuracy, which is indicated by a higher misalignment level. The accuracy issue is also visible in Figure 4, especially for the smaller coefficients from the tail of the echo path, but also for other peaks of the impulse response.

**Figure 5:**
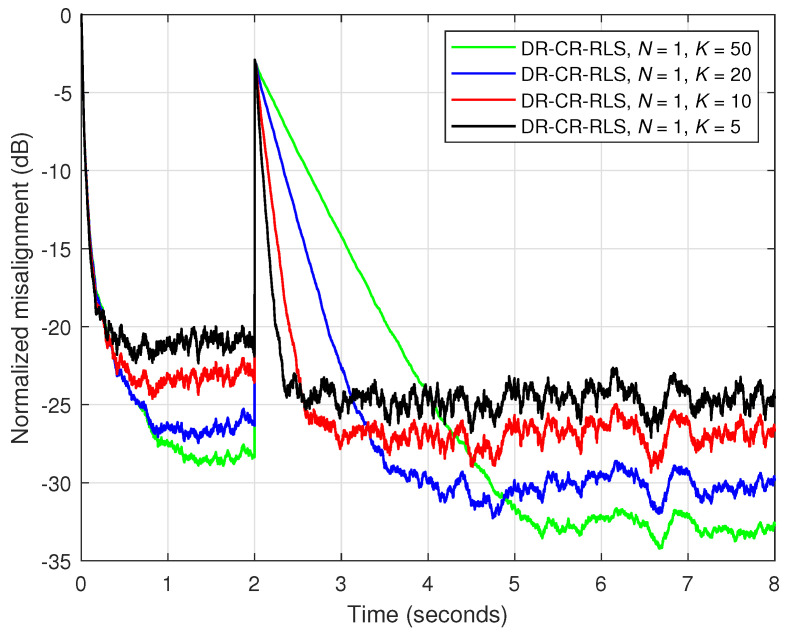
Normalized misalignment of the DR-CR-RLS algorithm using different values of the forgetting factor λ=1−1/(KL) and N=1 (equivalent to the conventional algorithm without data-reuse). The other conditions are the same as in Figure 3.

This behavior resembles the influence of the forgetting factor on the performance of RLS-type algorithms. In order to support this aspect, the previous experiment is repeated in Figure 5, but using different values of the forgetting factor (by varying the value of *K*) and setting N=1, which is equivalent to the conventional regularized RLS algorithm without data-reuse. As we can notice from Figure 5, a larger value of λ (or *K*) leads to a better accuracy of the estimate (i.e., lower misalignment), but pays in terms of the tracking behavior.

**Figure 6:**
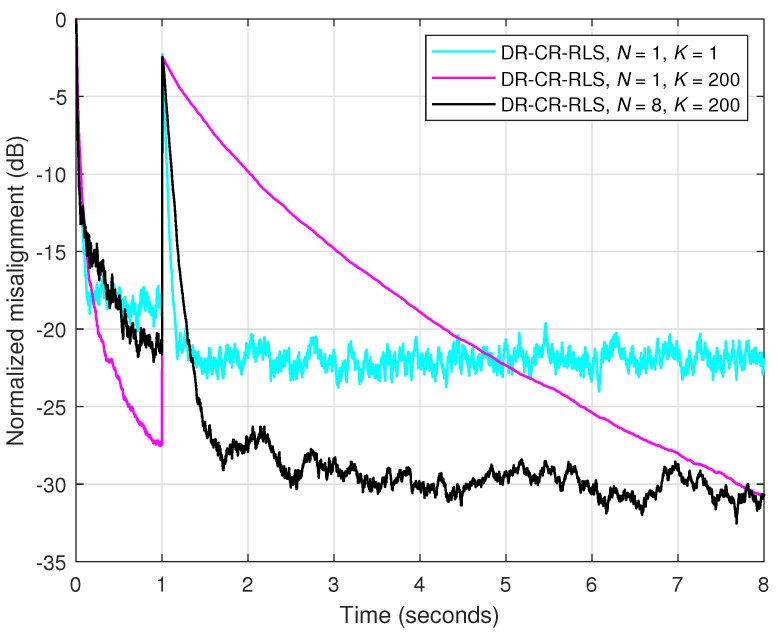
Normalized misalignment of the DR-CR-RLS algorithm using different values of the data-reuse parameter *N* and different values of the forgetting factor λ=1−1/(KL). The input signal is an AR(1) process, SNR=20 dB, L=128, $\delta =20\sigma_{x}^{2}$, and the echo path changes after 1 s.

This performance compromise can be better addressed by using a large value of the forgetting factor, while also increasing the value of *N*. This approach is supported in Figure 6, where the echo path change is introduced after one second. It can be noticed that the DR-CR-RLS algorithm using λ=1−1/(200L) and N=8 achieves a better compromise between the main performance criteria, as compared to the conventional regularized RLS algorithm (i.e., the DR-CR-RLS with N=1) using different forgetting factors. This flexibility of the data-reuse approach allows for a better control of the overall performance of the algorithm.

At this point, we should outline that the well-known affine projection algorithm (APA) [32] can be interpreted as a data-reuse LMS algorithm, since it acts based on an “optimal” reuse of data, through its projection order [24]. The update of APA is defined by the relation

(54) $$\begin{array}{cc} \hat{h}\left(n\right) & =\hat{h}\left(n-1\right)+\mu X\left(n\right){\left[\delta +X^{T}\left(n\right)X\left(n\right)\right]}^{-1}e\left(n\right), \end{array}$$

where μ is the so-called step-size parameter (with 0<μ≤1);

(55) $$\begin{array}{cc} X(n) & =\left[\begin{array}{cccc} x(n) & x(n-1) & \cdots & x(n-M+1) \end{array}\right] \end{array}$$

is the input signal matrix (of size L×M), with *M* representing the projection order; δ>0 is a regularization constant (that prevents a “bad” matrix inversion); and

(56) $$\begin{array}{cc} e(n) & =d\left(n\right)-X^{T}\left(n\right)\hat{h}\left(n-1\right) \end{array}$$

is the error signal vector, with

(57) $$\begin{array}{cc} d(n) & ={\left[\begin{array}{cccc} d(n) & d(n-1) & \cdots & d(n-M+1) \end{array}\right]}^{T} \end{array}$$

grouping the last *M* samples of the reference signal. The APA has a moderate computational complexity, which is proportional to O(ML). Note that the matrix inversion from (54) can be performed using different efficient techniques [33]. This algorithm also owns reliable convergence features, especially for correlated input signals, thus outperforming the LMS algorithm.

**Figure 7:**
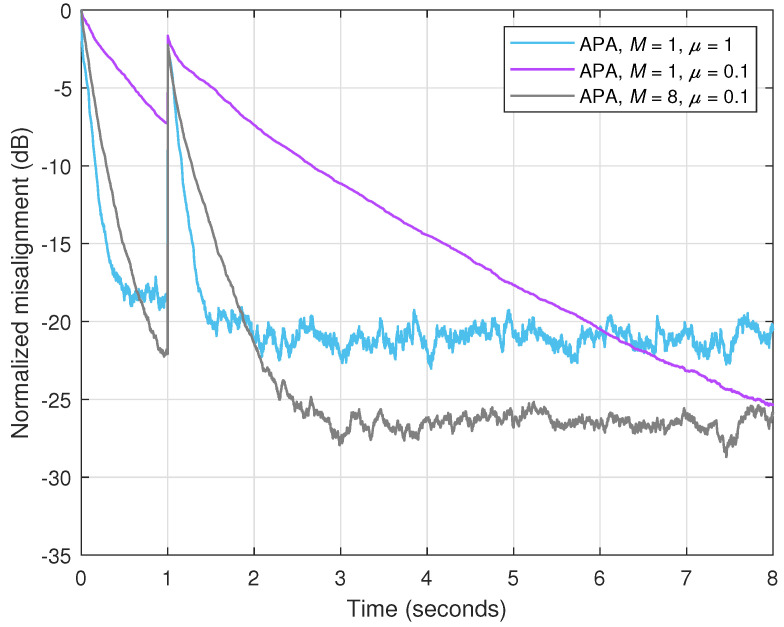
Normalized misalignment of the APA using different values of the projection order *N* and different values of the step-size μ. The other conditions are the same as in Figure 6.

In order to support its performance, as compared to the data-reuse regularized RLS-type algorithm, the experiment from Figure 6 is repeated in Figure 7, but using APA instead of the DR-CR-RLS algorithm. Two values of the projection order are used, i.e., M=1 and 8, together with two step-size parameters, μ=1 and 0.1. The APA using M=1 is equivalent to the well-known normalized LMS (NLMS) algorithm. Setting the value of the step-size parameter to μ=1 leads to the fastest convergence mode [3]. Similar to the combination of *N* and λ related to the DR-CR-RLS algorithm (see Figure 6), a performance balance can be achieved in case of APA by tuning the parameters *M* and μ, respectively. As we can notice in Figure 7, a higher value of *M* improves the convergence rate and tracking, but sacrificing the accuracy of the estimate, which is indicated by a higher misalignment level. On the other hand, a lower step-size parameter improves the accuracy, but pays in terms of the convergence features. Using a larger value of *M* together with a lower value of μ can lead to a better compromise between the performance criteria. Nevertheless, by comparing the results from Figure 6 and Figure 7, we can notice that the DR-CR-RLS algorithm outperforms the APA, achieving a faster initial convergence and a better tracking reaction, but also reaching a lower misalignment (i.e., better accuracy).

### 4.3. The DR-OR-RLS Algorithm

The DR-CR-RLS algorithm achieves a fairly reliable performance in good SNR conditions, as supported by the experiments provided in the previous subsection. However, in noisy conditions with low SNRs, the importance of using an appropriate regularization parameter becomes more significant. This aspect was also indicated in [18], where the regularization parameter of the data-reuse RLS algorithm was connected to the SNR. The resulting algorithm is referred to as DR-OR-RLS in Table 2 and its regularization parameter is denoted by $\delta_{o}$, considering that the value of the SNR is available. The experiments from this subsection outlines the importance of selecting a proper (constant) regularization parameter, as compared to an arbitrary one (based on a rule of thumb). The input signal remains the AR(1) process used in Section 4.2 and the forgetting factor is λ=1−1/(10L) for both the DR-CR-RLS and DR-OR-RLS algorithms, while the regularization constant of the DR-CR-RLS algorithm is the same as in the previous set of experiments (i.e., $\delta =20\sigma_{x}^{2}$).

**Figure 8:**
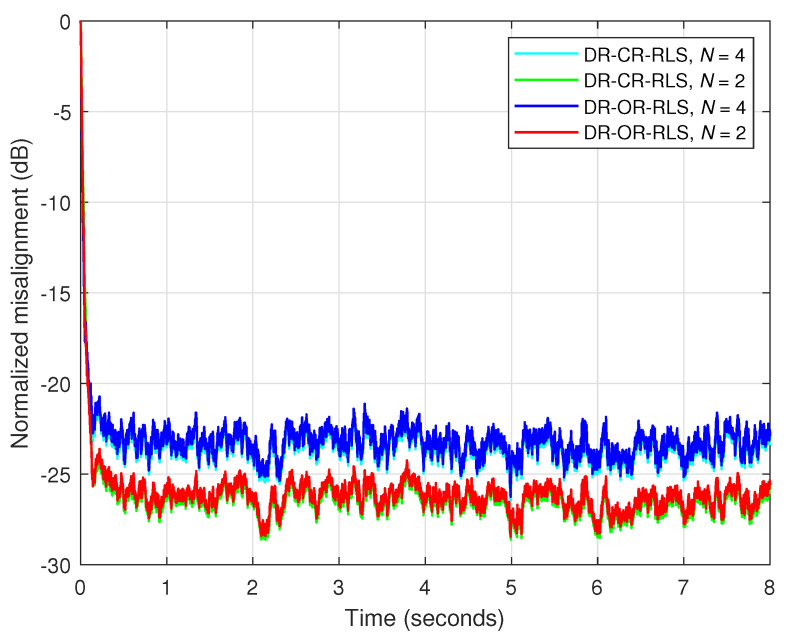
Normalized misalignment of the DR-CR-RLS and DR-OR-RLS algorithms using different values of the data-reuse parameter *N*. The other settings are λ=1−1/(10L), $\delta =20\sigma_{x}^{2}$ for DR-CR-RLS, and $\delta_{o}$ for DR-OR-RLS. The input signal is an AR(1) process, SNR=20 dB, and L=128.

**Figure 9:**
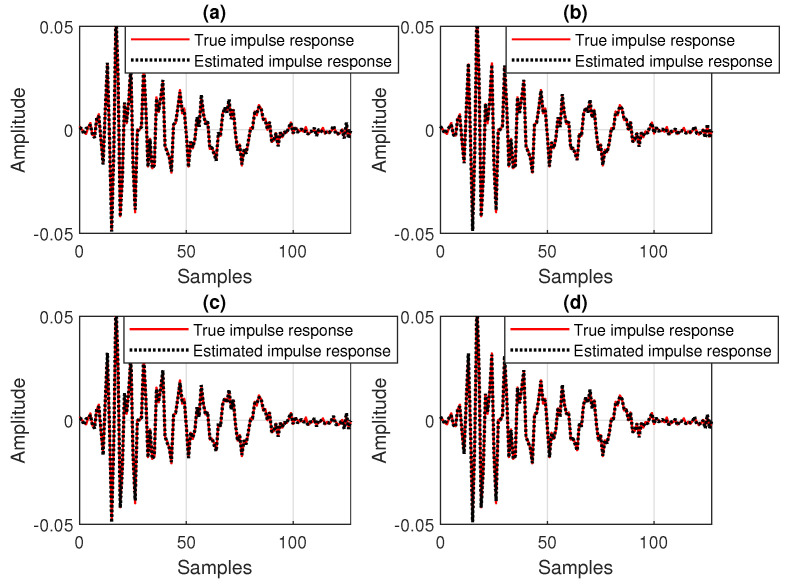
Estimated impulse response of the echo path, as compared to the true impulse response, for different values of the data-reuse parameter *N*, related to the experiment from Figure 8: (**a**) DR-CR-RLS with N=4, (**b**) DR-CR-RLS with N=2, (**c**) DR-OR-RLS with N=4, and (**d**) DR-OR-RLS with N=2.

First, in Figure 8, the SNR is set to 20 dB, so that the influence of the background noise is minor. As we can see, despite the value of the data-reuse parameter *N*, the behavior of the DR-OR-RLS algorithm is similar to its conventional DR-CR-RLS counterpart. In other words, the value of the regularization parameter does not influence the overall performance in this case (with good SNR). This aspect is also supported in Figure 9, where the estimated impulse responses are depicted (as compared to the true impulse response), for different values of *N*, related to the experiment from Figure 8. In all the cases, reliable estimates of h(n) are obtained due to the mild noisy conditions.

**Figure 10:**
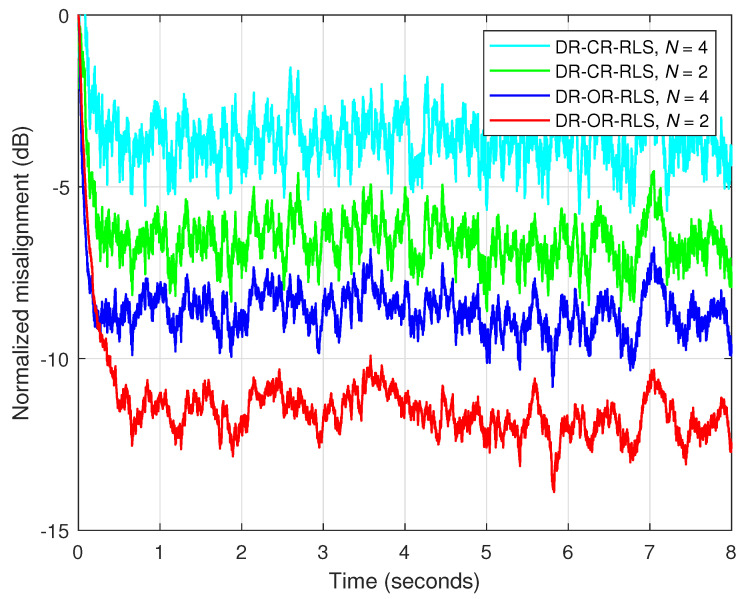
Normalized misalignment of the DR-CR-RLS and DR-OR-RLS algorithms using different values of the data-reuse parameter *N*, in a noisy environment, with SNR=0 dB. The other conditions are the same as in Figure 8.

**Figure 11:**
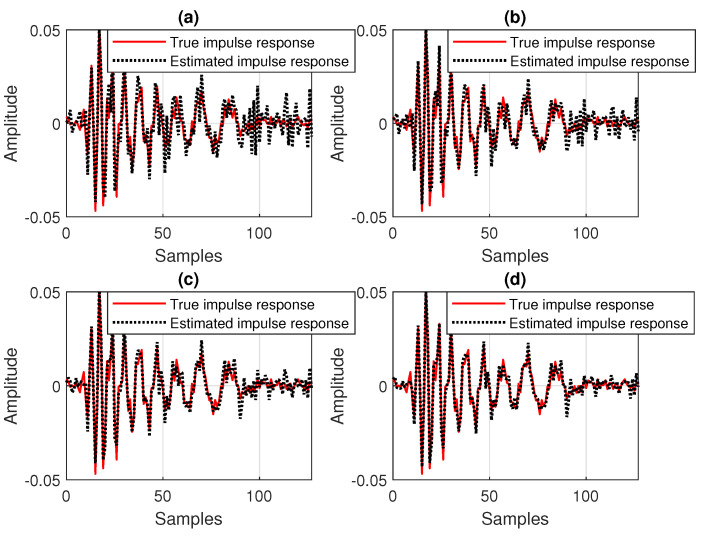
Estimated impulse response of the echo path, as compared to the true impulse response, for different values of the data-reuse parameter *N*, related to the experiment from Figure 10: (**a**) DR-CR-RLS with N=4, (**b**) DR-CR-RLS with N=2, (**c**) DR-OR-RLS with N=4, and (**d**) DR-OR-RLS with N=2.

Nevertheless, this behavior is no longer valid in low-SNR conditions, as supported in Figure 10. Here, SNR=0 dB, so that we deal with a very noisy environment. In this case, it can be noticed that the DR-OR-RLS algorithm that uses $\delta_{o}$ (which is related to the SNR) outperforms the conventional DR-CR-RLS benchmark, which is inherently limited by the value of its regularization constant ($\delta =20\sigma_{x}^{2}$). The difference is visible for both values of the data-reuse parameter (N=2 and 4) involved in this experiment. The DR-OR-RLS algorithm reaches lower misalignment levels as compared to the DR-CR-RLS version, which translates into better accuracy of the estimates. The estimated impulse responses from the experiment reported in Figure 10 are provided in Figure 11. Clearly, the challenging noisy conditions are influencing the accuracy of the estimates. However, the DR-OR-RLS algorithm leads to better estimates as compared to the DR-CR-RLS version, as indicated in Figure 10c,d, which support the results from Figure 10.

**Figure 12:**
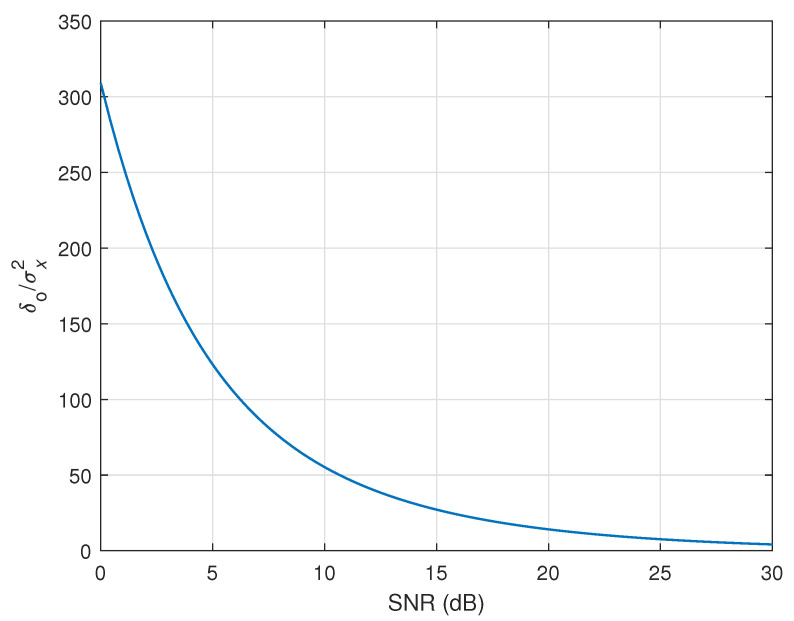
Evolution of the parameter $\delta_{o}/\sigma_{x}^{2}$ (related to the DR-OR-RLS algorithm) as a function of the SNR.

To support the previous discussion (and also the “rule of thumb” initially mentioned in Section 4.2), the evolution of the parameter $\delta_{o}/\sigma_{x}^{2}$ is depicted in Figure 12, with respect to the SNR. The normalization to $\sigma_{x}^{2}$ is just used to focus only on the component that depends on the SNR, within the regularization parameter $\delta_{o}$ of the DR-OR-RLS algorithm. As we can see from this figure, the lower the SNR, the higher the regularization parameter value should be. Also, when SNR=20 dB, the value of $\delta_{o}/\sigma_{x}^{2}$ is in the vicinity of 20, which justifies the rule of thumb selection ($\delta =20\sigma_{x}^{2}$) from Section 4.2.

### 4.4. The DR-VR-RLS Algorithm

The DR-OR-RLS algorithm from [18] can be considered as a theoretical benchmark for outlining the importance of selecting a proper value of the regularization parameter in different noisy conditions. Nevertheless, the true value of the SNR is not available in practice and should be estimated. This is performed within the DR-VR-RLS algorithm from Table 2, which uses the variable regularization (time-dependent) parameter $\delta^{\prime }\left(n\right)$, with the SNR estimated based on (17)–(19). In the following experiments, a speech sequence is used as input signal, which represents a challenge for the adaptive filtering algorithms, especially due to its nonstationary nature.

**Figure 13:**
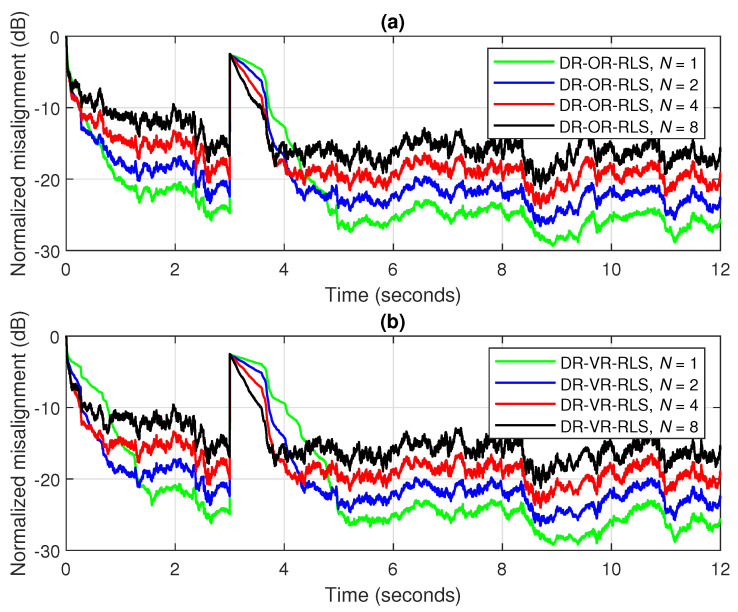
Normalized misalignment of (**a**) the DR-OR-RLS algorithm and (**b**) the DR-VR-RLS algorithm, using different values of the data-reuse parameter *N* and λ=1−1/(20L). The input signal is a speech sequence, SNR=20 dB, L=128, and the echo path changes after 3 s.

**Figure 14:**
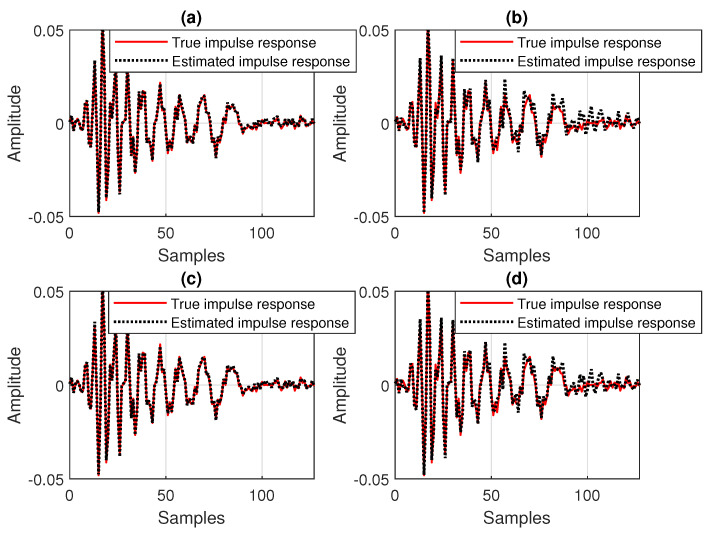
Estimated impulse response of the echo path (before the change), as compared to the true impulse response, for different values of the data-reuse parameter *N*, related to the experiment from Figure 13: (**a**) DR-OR-RLS with N=1, (**b**) DR-OR-RLS with N=8, (**c**) DR-VR-RLS with N=1, and (**d**) DR-VR-RLS with N=8.

A first comparison between the DR-OR-RLS and DR-VR-RLS algorithms is provided in Figure 13, for different values of the data-reuse parameter *N*, and using the forgetting factor λ=1−1/(20L). An echo path change scenario is considered after 3 s and the SNR is set to 20 dB. It can be noticed that the performances of these two algorithms are very similar under this scenario, proving that the estimate of the SNR used by the DR-VR-RLS algorithm is accurate. Only a slightly slower tracking reaction is noticeable as compared to the DR-OR-RLS algorithm, due to the evaluation of the estimates from (18) and (19), which are based on the exponential windowing technique using the forgetting factor λ (thus, resulting in an inherent latency). Related to this experiment, the estimated impulse responses for both algorithms (before the echo path changes) are provided in Figure 14, for two values of the data-reuse parameter, i.e., N=1 and 8. As expected, using a larger value of the data-reuse parameter results in a slightly lower accuracy of the echo path estimate, as indicated in Figure 14b,d. This behavior is valid for both versions of the data-reuse regularized RLS algorithm from Figure 13.

**Figure 15:**
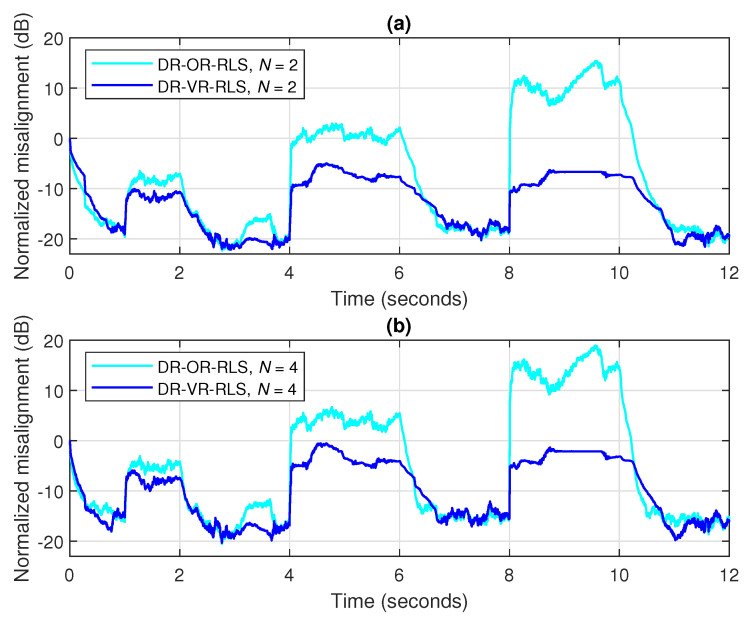
Normalized misalignment of the DR-OR-RLS and DR-VR-RLS algorithms using (**a**) N=2 and (**b**) N=4, in noisy conditions, considering three noise bursts with SNR=10 dB (between 1 and 2 s), SNR=0 dB (between 4 and 6 s), and SNR=−10 dB (between 8 and 10 s). The input signal is a speech sequence, SNR=20 dB (between the noise bursts), L=128, and λ=1−1/(10L).

The advantage of the practical estimation of the SNR within the DR-VR-RLS algorithm becomes more apparent in scenarios with variable background noise, as frequently happens in echo cancellation applications. Such a scenario is considered in Figure 15, where three bursts of white Gaussian noise of different durations corrupt the microphone signal (i.e., the acoustic sensor), with gradually decreasing SNRs, i.e., 10 dB, 0 dB, and −10 dB, respectively. Two values of the data-reuse parameter are used (i.e., N=2 and 4), while the forgetting factor is set to λ=1−1/(10L) for both algorithms. It can be noticed from Figure 15 that the DR-VR-RLS is more robust to the SNR variations, despite the increasing amount of noise. On the other hand, the DR-OR-RLS algorithm that uses the regularization parameter $\delta_{o}$ evaluated based on the steady-state background noise (i.e., between the bursts), with SNR=20 dB, is significantly affected in this scenario.

### 4.5. The DR-WR-RLS Algorithm

The DR-WR-RLS algorithm uses the regularization parameter $\delta^{\prime \prime }\left(n\right)$, which includes the NUR estimated based on (24) and (25), as also shown in Table 2. Besides the contribution of the external noise (related to its power estimate, $\sigma_{v}^{2}$), the NUR incorporates the model uncertainties, which are captured by the parameter $\sigma_{w}^{2}$. In this last subsection of simulation results, we compare the DR-WR-RLS algorithm with the previous two versions, i.e, the DR-OR-RLS and DR-VR-RLS algorithms, using a speech sequence as the input signal and different types of perturbation, in order to challenge the operating conditions.

**Figure 16:**
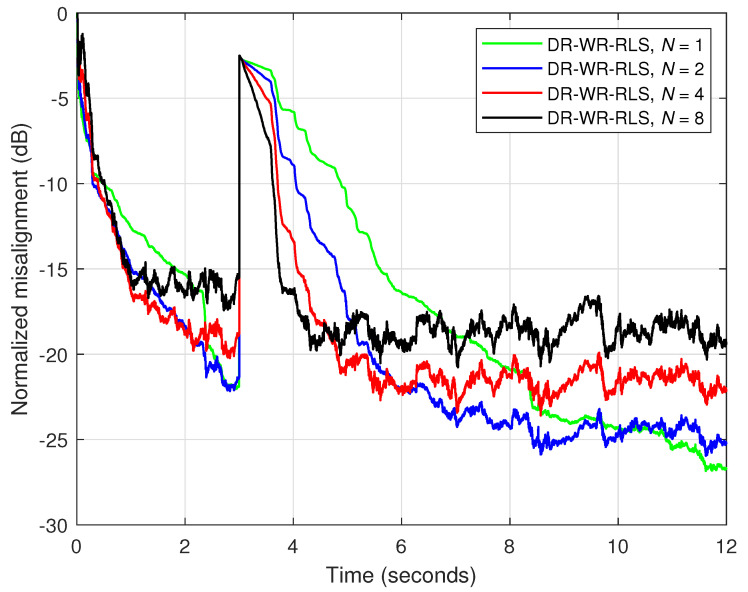
Normalized misalignment of the DR-WR-RLS algorithm using different values of the data-reuse parameter *N* and λ=1−1/(5L). The input signal is a speech sequence, SNR=20 dB, L=128, and the echo path changes after 3 s.

**Figure 17:**
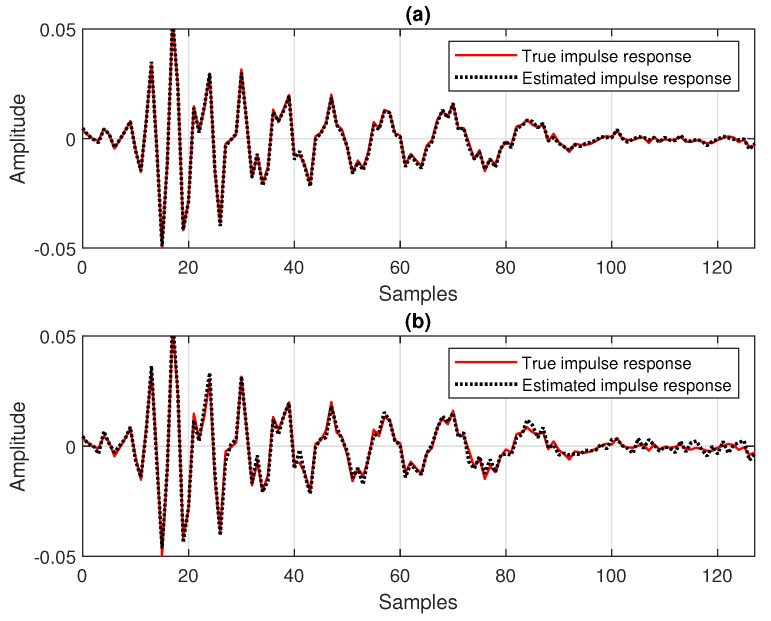
Estimated impulse response of the echo path (before the change), as compared to the true impulse response, for different values of the data-reuse parameter *N*, related to the experiment from Figure 16: (**a**) N=1 and (**b**) N=8.

First, the performance of the DR-WR-RLS algorithm is assessed for different values of the data-reuse parameter (*N*). An echo path change is introduced after 3 s and the forgetting factor is chosen as λ=1−1/(5L). The results are shown in Figure 16, where we can notice the same influence of the data-reuse parameter, i.e., faster convergence and tracking when *N* increases, at the cost of higher misalignment. Thus, a similar approach can be considered for a better compromise between the performance criteria: increasing the value of the forgetting factor together with the value of *N*. The estimated impulse responses provided by the DR-WR-RLS algorithm (before the echo path changes) are depicted in Figure 17a,b, for two values of the data-reuse parameter, i.e., N=1 and N=8, respectively. Here, a slight reduction in accuracy can be noticed for a larger value of *N*. This is an expected behavior that supports the results from Figure 16, which show that increasing the data-reuse parameter improves the tracking reaction but increases the misalignment level.

**Figure 18:**
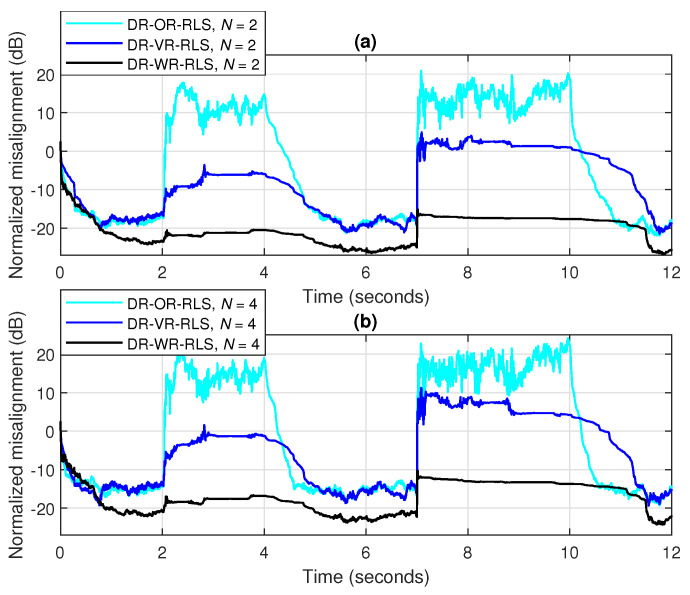
Normalized misalignment of the DR-OR-RLS, DR-VR-RLS, and DR-WR-RLS algorithms using (**a**) N=2 and (**b**) N=4, in noisy conditions, considering two bursts of highway noise (between 2 and 4 s) and engine noise (between 7 and 10 s). The input signal is a speech sequence, SNR=20 dB (between the noise bursts), L=128, and λ=1−1/(10L).

The main performance feature of the DR-WR-RLS algorithm is related to its robustness in different challenging scenarios. For example, in Figure 18, we consider a realistic communication scenario, when the acoustic sensor (i.e., the microphone) captures two different types of noise, for different periods of time. First, a highway noise appears between 2 and 4 s; then, an engine noise bursts between 7 and 10 s of the experiment. The background noise between these periods remains the same, with SNR=20 dB. The DR-OR-RLS algorithm used for comparison employs $\delta_{o}$, evaluated based on the SNR of this background noise, while the estimated (variable) SNR is used within the DR-VR-RLS algorithm. For all the algorithms, the forgetting factor is set to λ=1−1/(10L) and two data-reuse parameters are used, i.e., N=2 and 4. In both cases, it can be noticed that the DR-WR-RLS algorithm significantly outperforms its counterparts in terms of robustness to external perturbations. Even if the DR-VR-RLS algorithm is still better than the DR-OR-RLS version, it is also significantly affected during these challenging noisy conditions.

**Figure 19:**
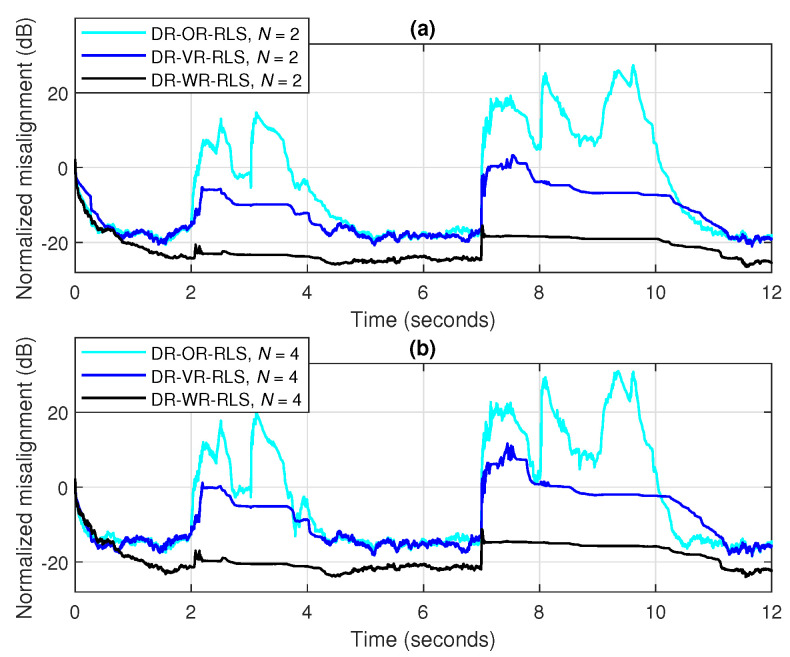
Normalized misalignment of the DR-OR-RLS, DR-VR-RLS, and DR-WR-RLS algorithms using (**a**) N=2 and (**b**) N=4, in double-talk conditions, considering two double-talk periods with different intensities, between periods of 2 to 4 s and 7 to 10 s, respectively. The input signal is a speech sequence, SNR=20 dB (for the background noise), L=128, and λ=1−1/(10L).

A similar experiment is shown in Figure 19, but considering the more challenging double-talk scenario [5]. In this case, the microphone signal captured by the acoustic sensor also contains the voice of the near-end talker, which acts like a large level of nonstationary disturbance for the adaptive filter. Two such double-talk periods are considered in this experiment, between periods of 2 to 4 s and 7 to 10 s, respectively, using different intensities, with the second one being longer in time and more intense in amplitude. Nevertheless, the DR-WR-RLS algorithm is still very robust under this difficult scenario, while the other two versions used for comparison are clearly disturbed and significantly biased during double-talk.

**Figure 20:**
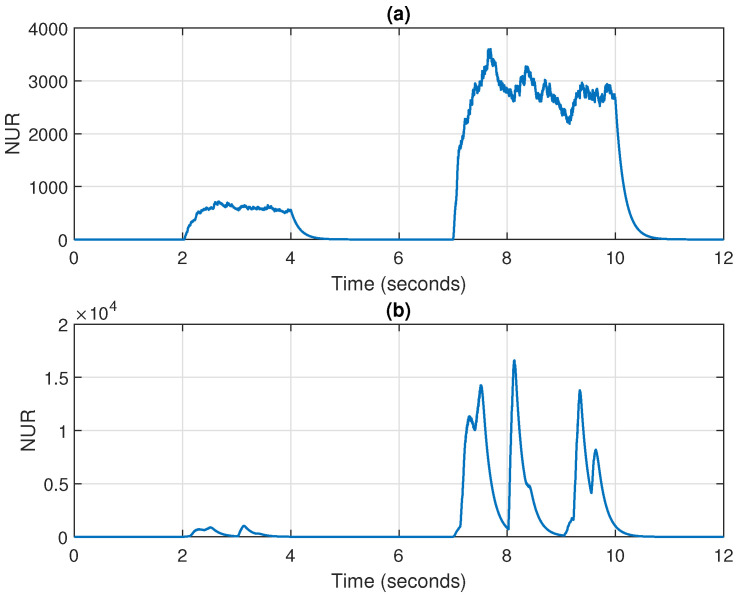
Time evolution of the NUR (related to the DR-WR-RLS algorithm) for the experiments reported in (**a**) Figure 18a and (**b**) Figure 19a.

In this framework, the estimated NUR of the DR-WR-RLS algorithm is depicted in Figure 20a,b, for the experiments from Figure 18a and Figure 19a, respectively (for N=2). In both cases, it can be noticed that this specific term provides a reliable measure related to the disturbance periods. Basically, the estimated NUR increases during these periods, thus reducing the update term of the DR-WR-RLS algorithm. As a result, its adaptation is slower and less affected by the disturbances, which represents the desired behavior in these challenging scenarios.

**Figure 21:**
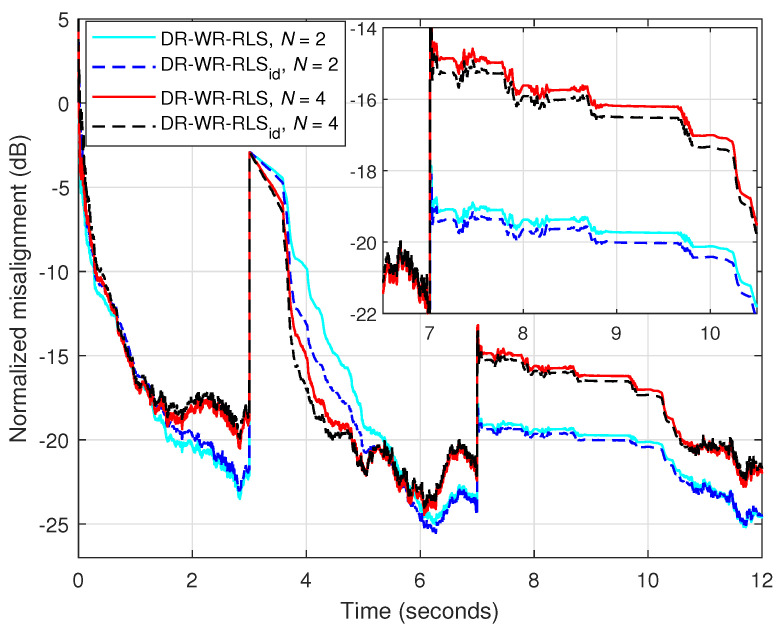
Normalized misalignment of the DR-WR-RLS algorithm and its “ideal” version (DR-WR-RLS_id_) using different values of the data-reuse parameter *N* and λ=1−1/(5L). The input signal is a speech sequence; SNR=20 dB (for the background noise), L=128, the echo path changes after 3 s, and a burst of engine noise appears between 7 and 10 s.

The overall performance of the DR-WR-RLS algorithm relies on the estimation of NUR, which depends on the estimated $\sigma_{v}^{2}$ and $\sigma_{w}^{2}$. Thus, a legitimate practical issue could be related to the sensitivity of the algorithm to this estimation, especially in challenging conditions like abrupt changes in noise or system dynamics. In order to assess such aspects, let us consider an “ideal” version of the algorithm, which is referred to as the DR-WR-RLS_id_, assuming that the near-end signal and the model uncertainties are available. This further allows the availability of a “true” NUR within the DR-WR-RLS_id_ algorithm. Under these circumstances, the experiment from Figure 21 considers an echo path change after 3 s, followed by a burst of engine noise that appears between 7 and 10 s (like in the second part of Figure 18). The input signal is a speech signal and the background noise is SNR=20 dB. The DR-WR-RLS algorithm is compared to its “ideal” version (DR-WR-RLS_id_) when using different values of the data-reuse parameter (N=2 and 4). The forgetting factor is set to λ=1−1/(5L) for both algorithms (with L=128). As expected, there is a slight delay in the tracking reaction of the DR-WR-RLS algorithm as compared to its “ideal” version, which becomes less apparent when the value of *N* increases. Also, in the “ideal” case, there is a minor improvement in terms of robustness during the noise burst. This is outlined in the zoom portion shown on the top-right of Figure 21. Nevertheless, the DR-WR-RLS algorithm is fairly reliable against the estimation of NUR, so that its sensitivity is quite minor. This represents an important practical aspect related to the identification of real-world time-varying systems, especially when operating in challenging conditions and environments.

### 4.6. Discussion

The results presented in Section 4.2, Section 4.3, Section 4.4 and Section 4.5 indicate several important performance features of the data-reuse regularized RLS algorithms, especially in terms of their robustness in noisy environments. While the forgetting factor is recognized as the main convergence parameter of the conventional RLS-type algorithms [1,2,3], tuning its value alone cannot always lead to a proper balance between the main performance criteria, i.e., convergence/tracking, accuracy, and robustness. Using a value of the forgetting factor close to its maximum bound results in a fast initial convergence rate and a good accuracy of the estimate, but with a slow tracking reaction when the impulse response of the system changes. Also, the robustness features of the algorithm are inherently limited when using only the forgetting factor as the control mechanism. In this context, a proper regularization term improves the robustness, while the data-reuse mechanism enhances the tracking capabilities even when using a large value of the forgetting factor. These are the main assets of the proposed data-reuse regularized RLS algorithms.

As shown in simulations, these algorithms can reliably operate in noisy conditions, with low SNRs. While the background noise inherently biases the accuracy of the estimate [5], the data-reuse regularized RLS algorithms can operate in noisy conditions, e.g., with SNR=0 dB, leading to a reasonable accuracy, with a misalignment level below −10 dB (as shown in Figure 10). This performance feature can be further improved when using a larger value of the forgetting factor. Moreover, with a proper (and practical) estimation of the regularization parameter, the data-reuse regularized RLS algorithms can fairly cope with challenging noisy bursts, e.g., when SNR=−10 dB (like in Figure 15). In addition, the DR-WR-RLS version outperforms its counterparts, being able to operate in adverse scenarios with nonstationary high-level background noises (as supported in Figure 18). In addition, it is also suitable for the double-talk scenarios, which are critical in echo cancellation applications (see Figure 19). All these characteristics support the potential practical applicability of the data-reuse regularized RLS algorithms, with appealing performances for real-world system identification scenarios.

The echo path models involved in this work originate from the ITU-T G168 Recommendation [31], for the sake of reproducibility of the results. Some of the noise sequences used in simulations are recorded in real environments, like the highway noise and engine noise (related to the results from Figure 18 and Figure 21). Also, several simulations were performed using a recorded female voice as the far-end (input) signal, like in Figure 13, Figure 14, Figure 15, Figure 16, Figure 17, Figure 18, Figure 19, Figure 20 and Figure 21. In addition, the near-end signal used in the double-talk scenarios from Figure 19 is also a recorded (male) voice. On the other hand, in most of the experiments, we used synthetic background noise, with different SNRs. In this context, it would be highly useful to test the algorithms with real-world acoustic data, for a more realistic experimental framework. This task represents a mandatory subject for future works, since the current paper basically outlines the main performance features of the data-reuse regularized RLS algorithms. The results have indicated improved robustness in different challenging scenarios (e.g., nonstationary noise and double-talk); however, there might be inherent limitations that could arise in practice, especially related to the practical implementation of these algorithms on DSP/FPGA platforms and the related numerical precision effects. Nevertheless, we aim to implement some of the challenging operations (like the matrix inversion) in a numerically robust manner, using computationally efficient iterative techniques, like the DCD method [28].

## 5. Conclusions and Perspectives

This paper has presented several regularized RLS algorithms that rely on the data-reuse method and own improved robustness features, in the framework of system identification. In this context, two regularization techniques have been involved, as presented in Section 2. The first one has led to a regularization parameter that depends on the SNR. The second one has also included the model uncertainties into the cost function, thus leading to a regularization parameter that includes the NUR. In addition, the data-reuse method applied to the regularized RLS-type algorithm has been formulated in a computationally efficient manner, using a single (equivalent) step for the entire data-reuse process, as developed in Section 3 (and summarized in Table 1). The resulting algorithms have been referred to as DR-CR-RLS, DR-OR-RLS, DR-VR-RLS, and DR-WR-RLS, as summarized in Table 2. The first two versions (i.e., DR-CR-RLS and DR-OR-RLS) represent theoretical benchmarks, using constant regularization parameters. The other two algorithms (i.e., DR-VR-RLS and DR-WR-RLS) are variable-regularized versions, since they use time-dependent regularization parameters and involve practical estimations of the SNR and NUR, respectively. As a result, they inherit the advantages of both the regularization-based approach and the data-reuse technique, leading to improved robustness and fast convergence/tracking, respectively. Simulation results obtained in the framework of echo cancellation (presented in Section 4) support these performance features. In this context, the algorithms have been tested in different challenging conditions, including variations of the SNR, different types of noise as external perturbations, and double-talk scenarios. Among the analyzed versions, the DR-WR-RLS algorithm stands out as the most performant one, especially in terms of its robustness against various adverse conditions.

In the future, we aim to further improve the DR-WR-RLS algorithm in terms of several aspects. First, the development of low-complexity versions of this algorithm is considered, based on the iterative techniques for solving systems of equations, like the CG and CD methods [28,29,30]. Second, the design of a multichannel version of the DR-WR-RLS algorithm represents a subject of interest, since there are different adaptive filtering applications that involve multiple acoustic sensors (i.e., microphones), for an enhanced listening experience. Third, tensor-based signal processing techniques could be used for the decomposition of the impulse response of the DR-WR-RLS adaptive filter, which would lead to improved overall performance due to a combination of filters with shorter lengths.

## Figures and Tables

**Table 1:** Data-reuse regularized RLS algorithm.

$\begin{array}{c} \underline{\mathrm{Parameters}:}\\ 0<\lambda \leq 1\;\left(forgetting\;factor\right)\\ N\geq 1\;\left(number\;of\;\mathrm{data-reuse}\;steps\right)\\ \underline{\mathrm{Initialization}:}\\ \hat{h}\left(0\right)=0_{L}\\ R\left(0\right)=0_{L\times L}\\ \underline{\mathrm{For}\mathrm{time}\text{-}\mathrm{index}}\;n=1,2,\ldots :\\ x\left(n\right)={\left[\begin{array}{cccc} x(n) & x(n-1) & \cdots & x(n-L+1) \end{array}\right]}^{T}\\ \hat{y}\left(n\right)=x^{T}\left(n\right)\hat{h}\left(n-1\right)\\ e\left(n\right)=d\left(n\right)-\hat{y}\left(n\right)\\ R\left(n\right)=\lambda R\left(n-1\right)+x\left(n\right)x^{T}\left(n\right) \end{array}$ Evaluation of δ, depending on the algorithm (see Table 2) $\begin{array}{c} P\left(n\right)={\left[R\left(n\right)+\delta I_{L}\right]}^{-1}\\ p(n)=P(n)x(n)\\ q\left(n\right)=x^{T}\left(n\right)p\left(n\right)\\ r(n)=1-q(n)\\ s\left(n\right)=\frac{1-r^{N}\left(n\right)}{q(n)}\\ \hat{h}\left(n\right)=\hat{h}\left(n-1\right)+s\left(n\right)p\left(n\right)e\left(n\right) \end{array}$

**Table 2:** Regularization parameters of the data-reuse regularized RLS algorithms.

$\begin{array}{c} \underline{\mathrm{DR}\text{−}\mathrm{CR}-\mathrm{RLS}\mathrm{Algorithm}:}\\ \delta =\mathrm{positive}\mathrm{constant}\\ \underline{\mathrm{DR}-\mathrm{OR}-\mathrm{RLS}\mathrm{Algorithm}:}\\ \mathrm{SNR}\;\mathrm{assumed}\mathrm{to}\mathrm{be}\mathrm{available}\\ \delta_{o}=L\frac{1+\sqrt{1+\mathrm{SNR}}}{\mathrm{SNR}}\sigma_{x}^{2}\\ \underline{\mathrm{DR}-\mathrm{VR}-\mathrm{RLS}\mathrm{Algorithm}:}\\ \sigma_{d}^{2}\left(n\right)=\lambda \sigma_{d}^{2}\left(n-1\right)+\left(1-\lambda \right)d^{2}\left(n\right)\\ \sigma_{\hat{y}}^{2}\left(n\right)=\lambda \sigma_{\hat{y}}^{2}\left(n-1\right)+\left(1-\lambda \right){\hat{y}}^{2}\left(n\right)\\ \mathrm{SNR}\left(n\right)=\frac{\sigma_{\hat{y}}^{2}\left(n\right)}{\epsilon +|\sigma_{d}^{2}\left(n\right)-\sigma_{\hat{y}}^{2}\left(n\right)|}\\ \delta^{\prime }\left(n\right)=L\frac{1+\sqrt{1+\mathrm{SNR}(n)}}{\mathrm{SNR}(n)}\sigma_{x}^{2}\\ \underline{\mathrm{DR}-\mathrm{WR}-\mathrm{RLS}\mathrm{Algorithm}:}\\ \sigma_{v}^{2}\left(n\right)=\lambda \sigma_{v}^{2}\left(n-1\right)+\left(1-\lambda \right)e^{2}\left(n\right)\\ \sigma_{w}^{2}\left(n\right)=\lambda \sigma_{w}^{2}\left(n-1\right)+\left(1-\lambda \right)\frac{{∥\hat{h}\left(n\right)-\hat{h}\left(n-1\right)∥}^{2}}{L}\\ \mathrm{NUR}\left(n\right)=\frac{\sigma_{v}^{2}\left(n\right)}{\epsilon +\sigma_{w}^{2}\left(n-1\right)}\\ \delta^{\prime \prime }\left(n\right)=\frac{1}{L(1-\lambda )}\mathrm{NUR}\left(n\right) \end{array}$

**Table 3:** Computational complexity of the main algorithms.

Algorithms	Number of Multiplications per Iteration
LMS	3L
RLS	$L^{2}+2L+O_{{•}^{-1}}$
DR-⋆R-RLS	$L^{2}+4L+N+O_{{•}^{-1}}+O_{\delta }$

## Data Availability

The original contributions presented in this study are included in the article. Further inquiries can be directed to the corresponding author.
